# Light-defined cold acclimation reprograms organ-specific metabolic and transcriptional landscapes for salt stress responses in maize

**DOI:** 10.1007/s00299-026-04008-z

**Published:** 2026-10-05

**Authors:** Kinga Benczúr, Fatemeh Gholizadeh, Magda Pál, Tibor Janda, Kamirán Áron Hamow, Mihály Dernovics, Katalin Nagy, Imre Majláth, Eva Darko, Jian Wu, Nhan Van Le, Gabriella Szalai

**Affiliations:** 1 Department of Plant Physiology and Metabolomics, Agricultural Institute, HUN-REN Centre for Agricultural Research, Brunszvik u. 2., 2462 Martonvásár, Hungary; 2 Beijing Key Laboratory of Development and Quality Control of Ornamental Crops, College of Horticulture China Agricultural University, Beijing, China; 3 Center for High Technology Research and Development, Vietnam Academy of Science and Technology, No.18 Hoang Quoc Viet Street, Cau Giay District, Hanoi City, Vietnam

**Keywords:** Cold acclimation, Gene expression, Light signalling, Metabolomics, Salt stress, *Zea mays* L.

## Abstract

**Key message:**

Cold acclimation of young maize plants induced a light-dependent, organ-specific reprogramming at the metabolomic and gene expression levels, which significantly altered responses to salinity.

**Abstract:**

It has been shown that light plays a decisive role in cold acclimation in plants. The core objective of the present work was to elucidate how light conditions during cold acclimation affect subsequent salt stress responses by co-regulating various processes among organs maize (*Zea mays* L.). Plants were cold-acclimated under sufficient light or shade and subsequently exposed to severe cold or salinity. Organ-specific analyses of leaves, stems, and roots combined chlorophyll fluorescence measurements with targeted and untargeted metabolomics, hormone and polyamine profiling, and stress-related gene expression were studied. Cold acclimation under sufficient light established organ-specific metabolic and transcriptional reprogramming that substantially altered subsequent salt stress responses. Light-acclimated plants showed coordinated, pathway-level metabolic adjustments across organs and distinct regulation of key stress-responsive genes, particularly in leaves and roots. Analyses indicated systemic, rather than isolated, metabolic rearrangements. These findings demonstrate that cold acclimation, depending on light intensity, establishes an organ-coordinated acclimated state that modulates subsequent salinity responses, highlighting the importance of environmental history and inter-organ regulation in stress adaptation. This study is the first to systematically reveal the organ-specific and systemic regulatory network underlying the light–temperature–salt interaction in maize.

**Graphical Abstract:**

**Figure:**
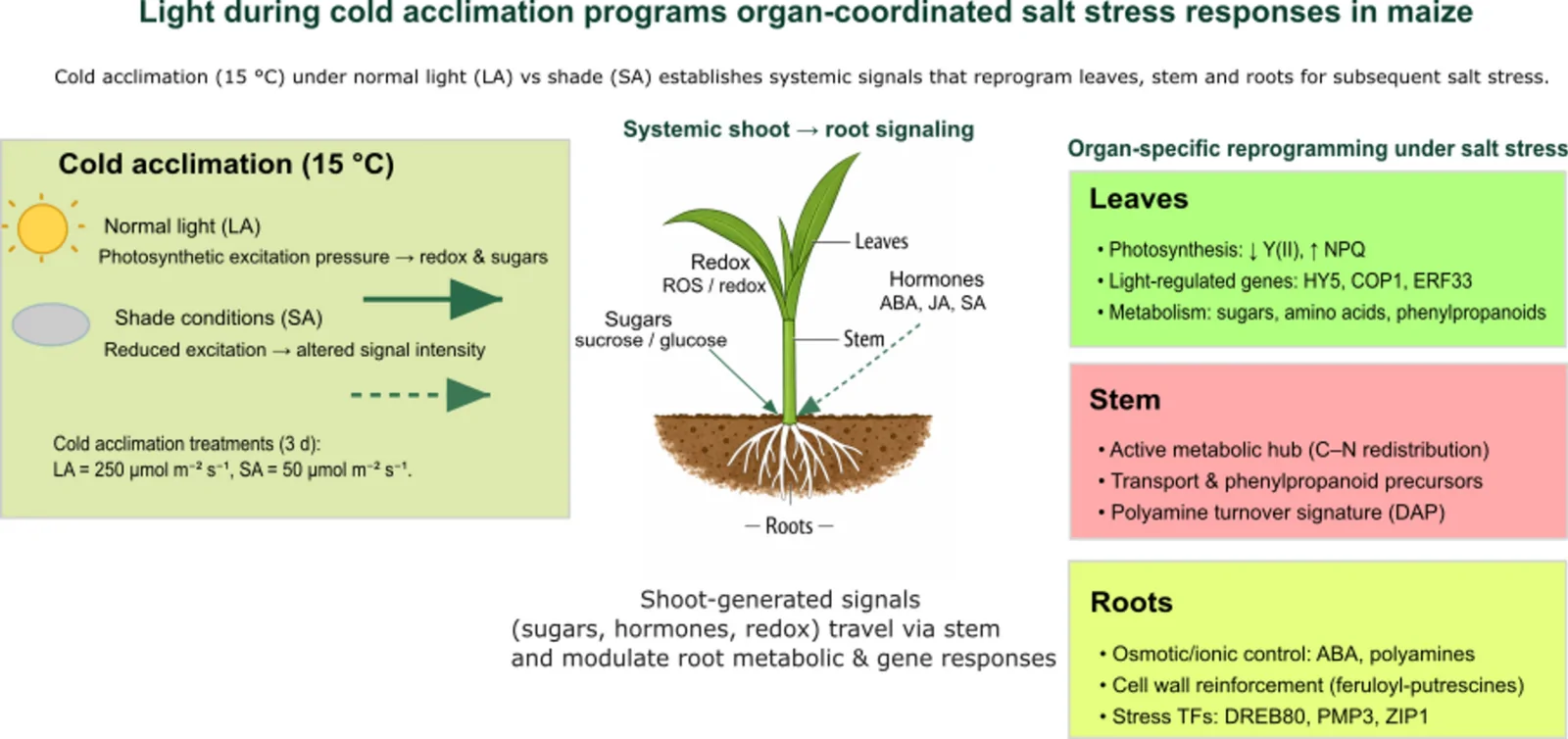

**Supplementary Information:**

The online version contains supplementary material available at https://doi.org/10.1007/s00299-026-04008-z.

## Introduction

Due to both global warming and intensive breeding improving the cold tolerance of modern maize hybrids, cultivation areas are continuously spreading northward. An important question in maize cultivation remains how maize genotypes can adapt to low temperatures, because despite general warming, sudden temperature drops may continue to occur. One of the simplest and most natural ways to increase cold tolerance is cold acclimation, which occurs when plants are exposed to specific periods of low, but non-lethal temperatures. Although maize, partly due to its C4 metabolism, is highly sensitive to cold, a certain degree of cold acclimation is possible even in this species (Janda et al. 1998; Szalai et al. 2018).

Cold acclimation of maize involves a coordinated set of multi-level responses, including gene regulatory, metabolic, cellular (membrane and cell wall), hormonal and physiological changes (Hamow et al. 2024). In typical cold-tolerant plants, these mechanisms operate efficiently, thus minimizing cell damage, maintaining proper water balance, stable photosynthesis and preserving growth capacity.

In maize, transcriptome analyses have found thousands of genes with differential expression in response to stresses (Szalai et al. 2018; Waititu et al. 2021). Among these, transcription factors—e.g., members of the MYB, ERF, NAC, WRKY, bHLH, MADS, etc. families—are common regulators of both development and stress responses in plants. The classical cold stress signalling pathway, the so-called ICE–CBF–COR (ICE: inducer of CBF expression, CBF: C-repeat binding factor, and COR: cold-regulated) pathway, is the central and best-understood transcriptional regulatory mechanism for cold stress acclimation. The activation of these three key genes by cold may lead to the subsequent induction of downstream cold-responsive genes (Zhou et al. 2022). Although the classical model of the ICE–CBF–COR pathway is known in various cold-tolerant plants, in maize, many such genes have been identified by cold induction, but relatively few of them were functionalized—so not all “cold-responsive” genes have been proven to cause actual acclimation. An important genetic factor is ZmRR1 (type-A response regulator). It regulates DREB1s and machinery related to cell wall synthesis (e.g., cellulose synthesis), thus contributing to cold resistance (Zeng et al. 2021). In maize, a gene encoding a key transcription factor (COLD-RESPONSIVE OPERATION LOCUS 1; COOL1) has recently been identified, some natural variants of which are associated with increased cold tolerance (Zeng et al. 2025). One of the negative regulators of this gene is the transcriptional factor ELONGATED HYPOCOTYL5 (HY5), which plays multiple roles in the regulation of stress responses (Xiao et al. 2022; Zhu et al. 2025).

Cold exposure alters hormonal homeostasis: e.g., stress hormones (such as abscisic acid) and other hormones (salicylic acid, etc.) may also play a significant role in the stress response (Ma et al. 2022). These hormonal signals may integrate the external cold stimulus into internal transcriptional, metabolic and physiological responses—e.g., abscisic acid-dependent metabolic and stress response pathways (Guo et al. 2021; Gholizadeh et al. 2025a, b).

In response to cold stress, cells usually accumulate more soluble sugars, amino acids, and other osmoprotectant compounds to preserve the cellular water and osmotic balance (Zhou et al. 2022). In addition, metabolic pathways—such as lipid, amino acid, and carbohydrate metabolism pathways are also modified during the cold acclimation periods. Cell wall stiffening and hardening may also be part of acclimation: some studies have reported an increase in the phenylpropanoid pathway and lignin/suberin synthesis in cold-tolerant lines—this serves to mechanically strengthen the cell wall (Zhou et al. 2026; Yu et al. 2022). The stability of the cell membrane and cell wall is critical: in cold, membrane permeability can be damaged, but in cold-tolerant lines, less membrane damage is observed. Cold stress often causes the accumulation of ROS (reactive oxygen species)—which, besides damaging lipid membranes, can also be harmful for proteins and DNA (Zhou et al. 2022). In maize, the increased activities of antioxidant enzymes may neutralize ROS. Cold-tolerant lines are able to protect certain photosynthetic proteins or regulate stomatal function—this helps minimize photo-oxidative damage.

However, not only temperature but also light affects frost resistance, as was demonstrated in cereals, such as rye and wheat (Gray et al. 1997; Apostol et al. 2006). Although light can cause photoinhibition at low temperatures, inhibiting the functioning of the photosynthetic electron transport chain, sufficient frost tolerance does not develop without adequate light intensity. The results showed that the overexcitation of the compounds in the photosynthetic electron transport chain serves as a signal to initiate certain defence processes that affect cold tolerance.

Even though light induces photoinhibition at low temperatures, thereby inhibiting the functioning of the photosynthetic electron transport chain, sufficient frost tolerance does not develop without adequate light intensity. The results suggest that overexcited electron transport is what serves as a signal to initiate certain defence processes that affect cold tolerance. In addition, light may play a role not only in cold tolerance, but also in cold acclimation processes enhancing the cold tolerance of maize during the low-temperature acclimation period. This led to the idea that photoinhibition is a necessary evil in the induction of cold tolerance (Szalai et al. 2018).

However, the effect of light on cold hardening affected not only in the leaves but also in the roots as reported by Szalai et al. (2018). Elevated sucrose and fructose accumulation was found in the roots during the cold hardening period in maize and the accumulation of these metabolites showed light-dependence (Szalai et al. 2018). Similarly, using the RNAseq and targeted, as well as untargeted analytical techniques, it has been presented that light conditions may affect certain stress responses in the roots of another cold sensitive crop species, rice plants, both at gene expression and metabolite levels (Gholizadeh et al. 2024). These results highlighted a few possible signalling pathways, for example, the NO-related mechanisms, which may have a special role in the shoot to root communication However, there are still many open questions about the light-regulated stress responses in the roots of the cold sensitive species. Based on our previous work (Szalai et al. 2018), a more in-depth study was conducted to further characterise the changes at metabolomics and gene expression levels in maize.

Plants exposed to a previous stress frequently exhibit altered responses to subsequent stress events. Depending on the persistence and nature of these responses, different concepts have been used to describe this phenomenon, including acclimation, priming, and stress memory. Acclimation refers to the physiological and biochemical adjustments that occur during exposure to a stress, whereas priming describes a state in which prior exposure enhances the responsiveness to a later stress. Stress memory generally implies the persistence of stress-induced information beyond the initial stimulus, resulting in modified responses upon subsequent stress exposure. Besides temperature stresses, high salinity also causes serious crop losses in various regions of the world. The core scientific objective of the present work is elucidating how light conditions during cold acclimation affect subsequent salt stress resistance by co-regulating responses among organs. According to our best knowledge, this study is the first to systematically reveal the organ-specific and systemic regulatory network underlying the light–temperature–salt interaction in maize. Therefore, in order to better understand the cross-talks between the effects of different stressors, we also investigated how the cold acclimation under different light conditions affects the stress responses at metabolite and gene expression levels to another important abiotic stressor, salinity. Therefore, in an additional experiment, subsequent high salt treatments were also applied. In this study, we tested the hypothesis that light conditions during cold acclimation modulate subsequent transcriptional and metabolic responses to salt stress in maize in an organ-specific manner. Specifically, we present that (i) cold acclimation under sufficient light induces changes in stress-responsive gene expression and metabolism, (ii) these changes differ between leaves, stem, and roots, and (iii) such light-dependent acclimation effects influence the plant’s response to a later osmotic stress, salinity.

## Materials and methods

### Plant materials and growth conditions

**Fig. 1:**
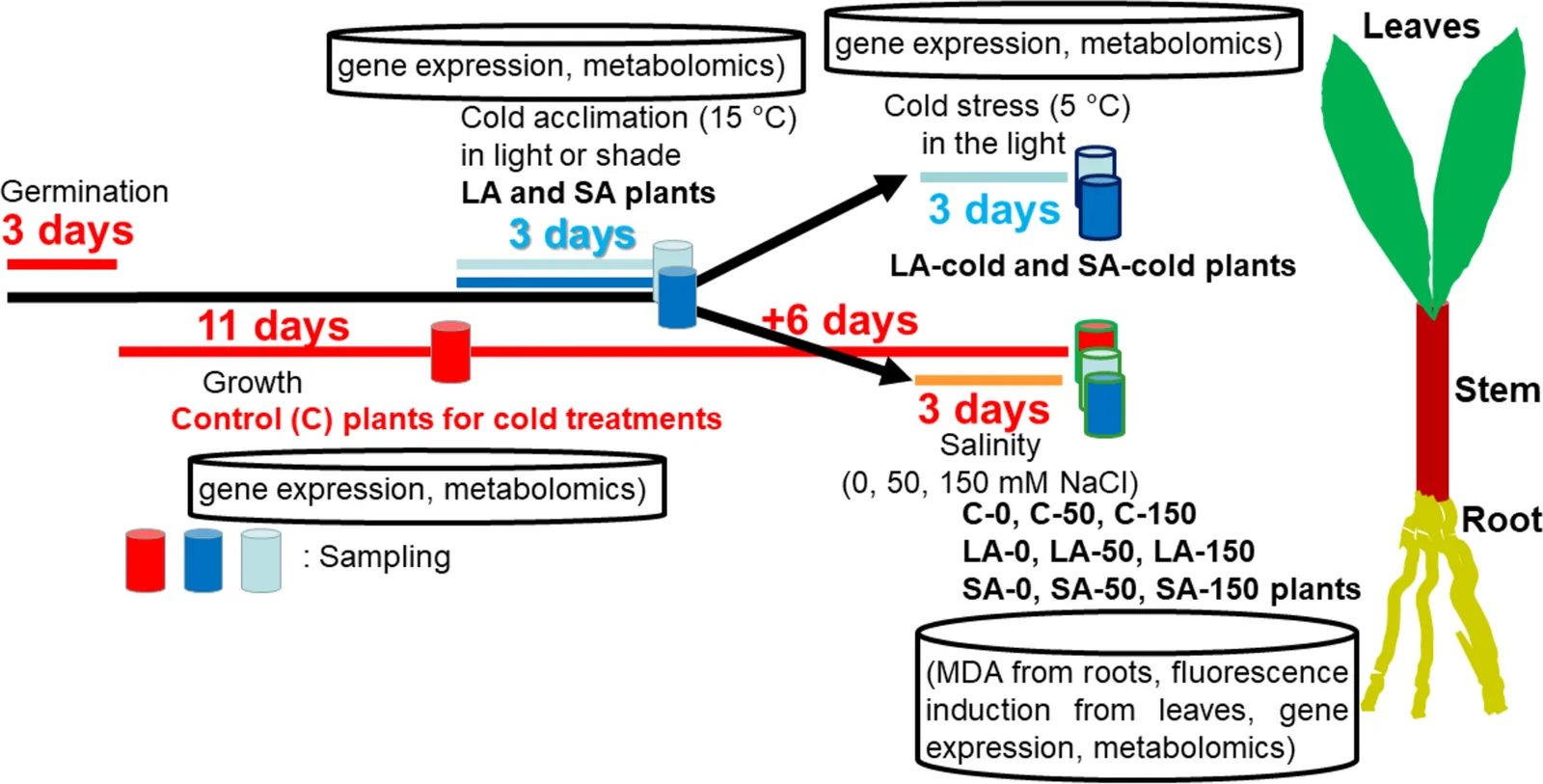
Schematic diagram showing plant growth and stress treatments. Control (C) represents plants at 22/20 °C before the cold acclimation period. LA and SA represent plants exposed to cold acclimation (at 15 °C) under normal light (LA, 250 µmol m^−2^ s^−1^) and shade conditions (SA, 50 µmol m^−2^ s^−1^). LA-cold and SA-cold represent light- and shade-acclimated plants after exposure to severe cold stress (5 °C) for 3 days, respectively. C-X, LA-X, SA-X represent plants exposed to X mM salinity for 3 days (X indicates 0, 50 or 150 mM salt treatments). Barrels show the time of sample collection. Samples were collected from the youngest fully developed leaves, stems, and roots. Details are found in the Materials and Methods section

The experiments were performed in the Agricultural Institute, HUN–REN Centre for Agricultural Research, Martonvásár, Hungary. The schematic description of the experiment is presented in Fig. 1. Maize seeds (*Zea mays* L. MV350 hybrid) were germinated for 3 days at 22 °C in wet filter paper. Then the seedlings were transferred to a hydroponic system (1 L beakers with 250 ml of modified Hoagland solution), as described by Pál et al. (2005). The young plants were grown for 11 days under controlled conditions in a Conviron PGV-36 growth chamber (Controlled Environments Ltd, Winnipeg, MB, Canada) at 22/20 °C day/night temperature with 16/8 h day–night cycle at a photosynthetic photon flux density (PPFD) of 250 µmol m^−2^ s^−1^. We initially started with 96 beakers, including initially 6 germinated seeds. For getting homogenous plant material, the too small or big plants were eliminated at their early growth stage. After 2 weeks from the beginning of the germination, plants were transferred to a low, acclimating temperature (15 °C) either at the same growth PPFD (LA: light-acclimated plants), or at reduced light conditions (50 µmol m^−2^ s^−1^; SA: shade-acclimated plants). After 3 days, certain plants were exposed for 3 days to either severe cold stress (5 °C, continuous light; without precooling the hydroponic solution) or to high salinity, meaning exposed to 0, 50, or 150 mM NaCl. Samples were collected from different parts of the plants (youngest fully expanded leaves, stem and root) for gene expression, biochemical and metabolomics analyses. Samples for cold treatment were collected just before the acclimation (control), after 3 days of cold acclimation (LA and SA) and then after 3 days of severe cold treatment at 5 °C (LA-cold and SA-cold). For investigating the combined effect of cold acclimation and salt stress after the third day of the salt treatment samples were collected from non-acclimated (Control 0, 50 and 150 mM), and either in the light (LA 0, 50 and 150 mM) or in the shade (SA 0, 50 and 150 mM) cold-acclimated plants. We collected a number of the youngest, fully developed leaves, stems, and roots in five biological replicates (approximately 5–6 g each). We then pulverized the samples in liquid nitrogen, weighed out the appropriate amounts of the resulting homogeneous powder for each analysis, and stored them at − 80 °C.

### Chlorophyll-a fluorescence induction analysis

The fluorescence induction analyses were carried out using a pulse amplitude modulated fluorometer (PAM) with IMAG–MAX/L (*λ*=450 nm) unit (Imaging-PAM MSeries, Walz, Effeltrich, Germany). The maximum quantum yield (F_v_/F_m_) was determined after 30 min dark adaptation on the youngest fully expanded leaves. The saturating light was 3000 µmol m^−2^ s^−1^ using 0.8 s duration. Photosynthesis was then induced by using 250 µmol m^−2^ s^−1^ actinic light intensity for 7 min and the Y(II) (actual quantum yield of PSII), Y(NPQ) (quantum yield of regulated non-photochemical energy loss) and Y(NO) (quantum yield of non-regulated non-photochemical energy loss) parameters were determined at the end of light treatment based on Klughammer and Schreiber (2008).

### RT–qPCR

Total RNA was extracted from maize leaf, stem, and root tissues for each treatment using TRIzol reagent, following the manufacturer’s guidelines. RNA concentrations were determined using a NanoDrop 2000c spectrophotometer (Thermo Scientific, USA). The RNA samples were then treated with DNase I and purified with the Direct-zol^™^ RNA MiniPrep Kit (Zymo Research, Irvine, CA, USA) according to the provided protocol. Complementary DNA (cDNA) synthesis was performed using M-MLV reverse transcriptase (Promega Corporation, Madison, WI, USA). For RT–qPCR, the *ZmEF1a* gene was used as the reference gene. In preliminary tests, RT–qPCR analysis was carried out with four reference genes, *ZmEF1a*, *ZmActin1*, *ZmUbi2* and *ZmTubulin*. The reference gene *ZmEF1a* has been validated as stable under different conditions and it was more stable than others; therefore, this was selected for the comparisons. Quantitative PCR was conducted with PCRBIO SyGreen Mix (PCR Biosystems, London, UK) on a CFX96 Real-Time PCR Detection System (Bio-Rad, Hercules, CA, USA). Each treatment was analysed with three biological replicates and three technical replicates, using gene-specific and housekeeping primers listed in Table 1. Relative gene expression levels were determined using the 2^−ΔΔCT^ method (Livak and Schmittgen 2001).

**Table 1:** Genes and primers used for RT–qPCR analyses in the present study

Genes	Reverse primer (5'–3')	Forward primer (5'–3')	Reference
*ZmEF1a*	TGGGTCCTTCTTCTCCACAC	TCTCAAGAACGGTGATGCTG	(Majláth et al. 2022)
*ZmHY5*	GCTGTCTGACCCCAGTTTTTG	CCGACAGATACTGAAGAACA	(Huai et al. 2020)
*ZmCOP1*	ATCTGCAAAACTTCATTCCCTAACC	CTAACAGTCAGGGGACCATAAAAG	(Huai et al. 2020)
*ZmERF33*	CATGGTTTTCTCTGACGTTCTC	*CACCCAACACATACAAACACAC*	(Cheng et al. 2023)
*ZmPMP3-1*	GGCGTAGATGGCAGTGATGA	CAACTGCGTGGACATCCTGA	(Fu et al. 2012)
*ZmSWEET1a*	CGATAGGAGGCAGTTGAGC	CAGGTTCTTCTTCGGAGTTT	(Zhu et al. 2022)
*ZmPMP3-5*	GATTTCTTCAGCCGAGCAAACT	TTCTGGATCTGCCTTATCCTCAC	(Fu et al. 2012)
*ZmbHLH95*	CTCCTTCAGGTACTCGATTGC	GAAGCTTAATGACCGGCTCTA	(Zhang et al. 2018)
*ZmDREB80*	GCGCATAAACATAGGAACACAT	*TGCTCTCTTCTTTAGTTGTGGT*	(Cheng et al. 2023)
*ZmZIP1*	TTGATGGTTGTTTTCTGGTCGT	CCTCTCTGCGTTGGTTGCTCT	(Xu et al. 2021)
*ZmPIF1*	TCCTCGCATCCCACCAGAC	GCAGTCGCTACTCCATCGC	(Gao et al. 2019)
*ZmCOLD1*	GTGGTACAAAGAATGGGTCAAC	ACAAGCATCCGATTGATTGATG	(Jin et al. 2021)
*ZmPAO1*	GTCGACGATCTTTCCAGACTTA	GTGGACTACTACAAGTTCGACT	(Xi et al. 2022)

### Primary metabolism analyses on GCxGC TOF MS

For the analysis of sugars, amino acids and organic acids analysis, sample preparation, derivatization and GCxGC TOF MS measurement were performed according to Gondor et al. (2021) using 200 mg plant material. The internal standard (Adonitol (1 mg mL^−1^) from Merck KGaA, Darmstadt, Germany) was added to leaf, stem and root samples, which were then extracted methanol solutions (60% and 90%). Aliquot of the supernatant was dried under vacuum. The dried samples were derivatized and then transferred to the LECO Pegasus 4D GCxGC TOFMS (LECO Corporation, St Joseph, MI, USA). The primary column was Rxi-5MS 30 m length and secondary column was Rxi-17Sil MS 1,5 m length (Restek Corporation, Bellefonte, PA, USA). Data evaluation was performed with LECO ChromaTOF 4.72 (LECO Corporation, St Joseph, MI, USA), and identification was based on the Kovats retention index, NIST Mass Spectral Search Program (InChI Library v 1.05.(NIST, Gaithersburg, MD, USA) and Leco-FiehnLib (LECO Corporation, St Joseph, MI, USA) library, as well as standards.

### Sample preparation for untargeted and targeted LC/MS analyses

Sample preparation was performed according to Pál et al. (2019) with minor modifications. Briefly, 0.2 g of plant material was extracted with 2 × 1 mL methanol:water (2:1, v/v) containing 20 ng mL⁻^1 2^H₆-cis,trans-abscisic acid (OlChemIm s.r.o., Olomouc, Czech Republic) as an internal standard. All solvents and chemicals used for sample preparation and analysis were of UPLC gradient or GC Pestinorm Supratrace grade (VWR, Radnor, PA, USA). Samples were extracted twice by vigorous vortex mixing and shaking in a Spex MiniG 1600 homogenizer (Metuchen, NJ, USA) using a cryo-cooled rack at 1250 rpm for 3 min. Following centrifugation at 16,500 × g for 10 min at 4 °C, the supernatants were collected and pooled. One millilitre of the combined methanol–water extract was subjected to liquid–liquid partitioning with 0.5 mL n-hexane to remove carotenoids and lipids. After centrifugation at 10,000 × g for 10 min at 4 °C, the methanol–water phase was recovered and filtered through 0.22 μm PTFE syringe filters prior to analysis.

### Untargeted liquid chromatography–mass spectrometry (LC–MS) analysis

Untargeted LC–MS analysis was carried out as it was described earlier (Szalai et al. 2022). Briefly, leaf samples were ground in liquid N_2_, and 0.2 g subsamples were weighed into 2.0 ml safety Eppendorf tubes with the addition of 1.0 ml of 40% (*v*/*v*) methanol. After vortex mixing, the tubes were inserted into a pre-cooled (− 18 °C) tube holder, and the extraction was carried out in a 1600 MiniG^®^—Automated Tissue Homogenizer and Cell Lyser (SPEX; Rickmansworth, UK) for 3 min at 1500 rpm. The samples were then centrifuged at 15 000×*g* for 15 min at +4 °C, after which the supernatants were recovered and placed in separate Eppendorf tubes. The residues were re-extracted with 1.0 ml of 90% (*v*/*v*) methanol in a MiniG® instrument (3 min), followed by centrifugation as described above. Aliquots of the combined supernatants were 1 + 2 (*v* + *v*) diluted with water containing 0.15% (*v*/*v*) formic acid. After dilution, the sample solutions were filtered through 0.22 μm pore-sized disposable PTFE syringe filters and injected into the LC–MS setup. A Vion ion mobility quadrupole time-of-flight mass spectrometer (UPLC–IMS–QTOF; Waters) with a Z-spray ion source was applied both in positive and negative ionization modes, either with MS^E^ or with MS^E^–MSMS/DDA functions. Chromatographic elution was provided by an Acquity UPLC I-Class system (Waters) using a BEH-C_18_ reversed phase (RP) UPLC column (100 mm × 2.1 mm × 1.7 μm; Waters). The instrument was controlled using UNIFI software (version 1.9.4; Waters). The related instrumental parameters are described in Supplementary Table S1.

Multivariate statistical analyses were carried out according to Szalai et al. (2022) and only major modifications are presented here. In brief, the Vion UNIFI LC–MS data were processed and normalized with the Progenesis QI (Version 27.26.1020; Nonlinear Dynamics, Quayside, Newcastle Upon Tyne, UK) and EZinfo (version 3.0.3; UMetrics AB, Umeei, Sweden) software packages. Molecular entities assigned in the negative and positive ion modes were subjected to ANOVA and Principal Component Analysis (PCA). For group difference calculations, the PCA data were exported to EZinfo, where pairwise group differences were visualized in VIP-plot arrangement. Data (= compounds) were considered significant and transferred back to Progenesis QI for identification if the VIP values were higher than 2.0. Compounds found to be significant in group difference analyses in either ionization mode were sorted by their maximum abundance, and entries exceeding 1500 cps, i.e., the experimentally determined limits for reliable MS/MS acquisition, were individually and manually checked for redundancies (e.g., because of incorrect isotopologue alignment) in the LC–MS spectra. Non-redundant entries were subjected to directed data acquisition (DDA)–MS/MS fragmentation in both negative and positive modes when applicable.

The following settings were applied for compound identification: precursor tolerance: 5 ppm; fragment tolerance: 10 ppm; isotope similarity: 95%; elemental composition: H 0–180, C 0–100, N 0–10, O 0–100, P 0–3, S 0–4. The following data sources were considered: Biosynth Carbosynth, ChEBI, ChEMBL, Extrasynthese, FooDB, HMDB, KEGG, LipidMAPS, MassBank, Molbank, Nature Chemistry, Nature Chemical Biology, NIST, NIST Chemistry WebBook, Phenol Explorer, and PlantCyc. The ultimate or tentative identification and assignment of statistically differentiating components were based on either the use of the commercially available authentic standards (feruloyl-putrescine (/>97% purity; BOC Sciences, Shirley, NY, USA/; neochlorogenic acid/>98%; Merck-Sigma Group, Darmstadt, Germany/; and rutin trihydrate/>94.0%; Merck-Sigma Group/), or their accurate mass, MS/MS fragments and chromatographic elution order according to Montone et al. (2021), Pál et al. (2025), Mravčáková et al. (2019), Lourenço et al. (2021), Othibeng et al. (2021), and Marczak et al. (2016) (Supplementary Table S2).

### Plant hormones, related substances and phenolics

Chromatographic analysis was performed using a Waters Acquity I-Class UPLC system equipped with a PDA detector and coupled to a Xevo TQ-XS triple quadrupole mass spectrometer with a UniSpray^™^ (US) ion source (Waters Corp., Milford, MA, USA). Separation was achieved on an HSS T3 column (1.8 μm, 100 × 2.1 mm). The mass spectrometer was operated in multiple reaction monitoring (MRM) mode according to Pál et al. (2019). Detailed UPLC–MS conditions, method parameters, and MRM transitions used for quantification are provided in Supplementary Table S3.

### Polyamine determinations

For polyamine analysis, the sample preparation, derivatization, and HPLC analysis and detection were carried out according to Pál et al. (2022). Briefly, 200 mg of plant tissue was homogenized with liquid N_2_ in a mortar and further grinded with 2 ml of 0.2 N HClO_4_. The homogenates were centrifuged at 4 °C for 10 min at 10,000 g. For dansyl chloride derivatization reactions, 100 μl supernatant and 200 µl of saturated sodium carbonate were used, with 400 μl of dansyl chloride in acetone (5 mg ml^−1^). The reaction mixture was incubated at 60 °C for 1 h in the dark, and stopped with 100 μl of proline (100 mg ml^−1^). After further incubation at room temperature in the dark for 30 min, the polyamines were extracted with 500 μl of toluene by vigorous vortexing for 30 s. The organic, upper phase, containing the polyamines, was removed and completely dried. The residue was dissolved in 0.5 ml of methanol, and after ultrafiltration through nylon membranes, was used for HPLC analysis. The 1,3-diaminopropane (DAP), putrescine (PUT), spermidine (SPD) and spermine (SPM) contents were determined after separation on via HPLC using a W2690 separation module on a reverse phase column (Kinetex C18, 5 μ, 100 × 2.1 mm, Phenomenex, Inc.) and detection with a W2475 scanning fluorescence detector (excitation at 340 nm and emission at 515 nm) (Waters, Milford, MA, USA).

### Statistical analyses

The results are the means of five biological replicates for chlorophyll-a fluorescence induction measurement, and at least three biological replicates for chromatographic determinations. All reactions for gene expression analyses were performed in triplicate, including three biological replicates and three technical replicates. Mean comparisons among treatments were conducted using the least significant difference (LSD) test at *P* < 0.05. GraphPad Prism (version 9.0.1) was employed to create visual representations of the data associated with gene expression patterns. The data were statistically evaluated using the standard deviation in Microsoft Excel (STDEV.S function) with *n* ≥ 3. Different letters indicate statistically significant differences (*p* < 0.05) between multiple groups (one-way ANOVA with Tukey’s post hoc test was performed using SPSS 16.0). The PCA was carried out in the ORIGIN (ver. 2023b).

## Results

### Physiological characterisation of salt-treated plants with or without previous exposure to cold acclimation

**Fig. 2:**
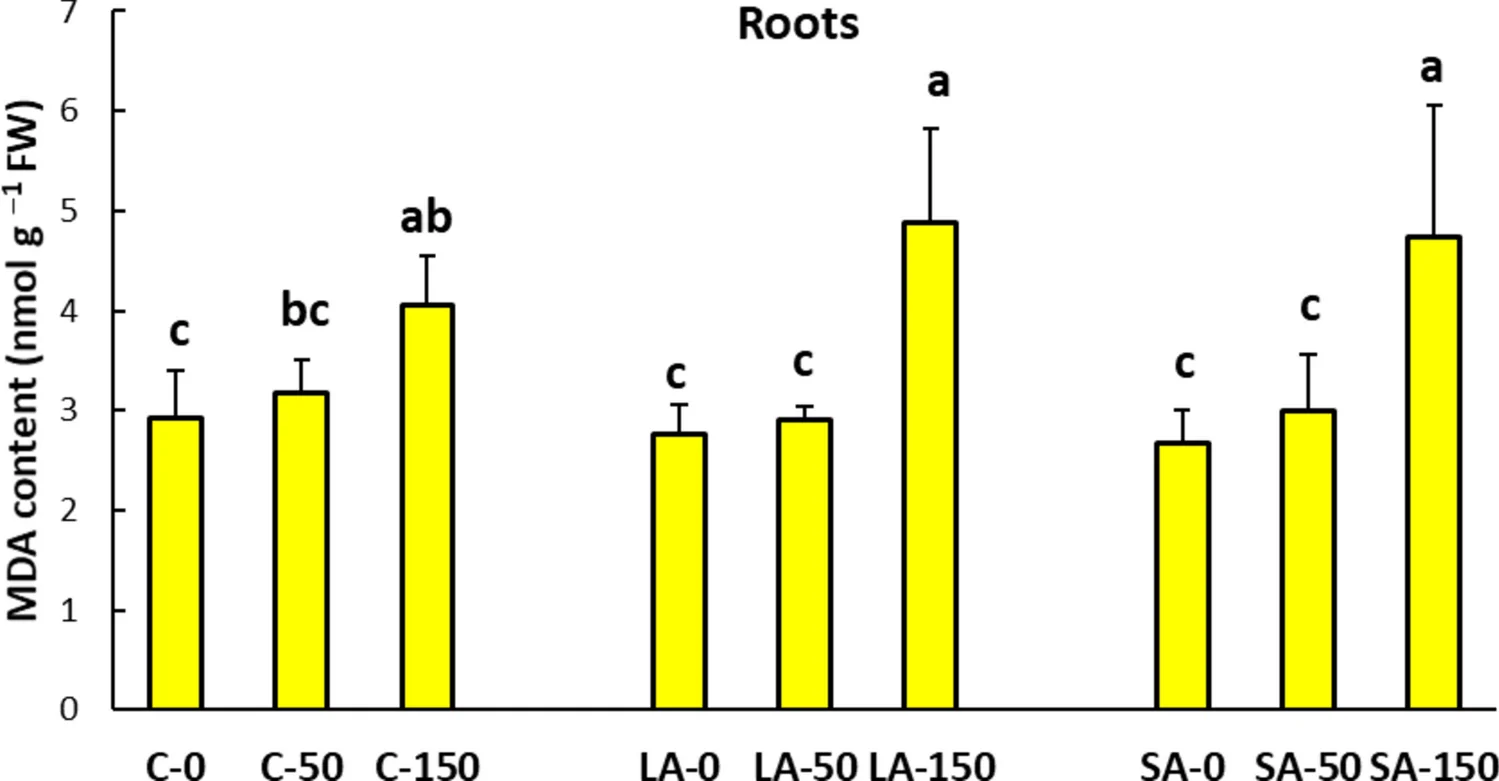
Changes in the MDA levels in the roots of maize plants after exposure to 0, 50, or 150 mM NaCl with or without previous cold acclimation under different light conditions. *C* control, no cold acclimation, *LA* cold-acclimated at growth light, *SA* cold-acclimated in the shade. Mean and SD values, *n*=5. Different letters indicate significant differences at *p* < 0.05 level

In the present work, young maize plants were exposed to cold acclimation temperature (15 °C), which was followed by either a subsequent severe cold (5 °C) or by different levels of salt stress. The effects of low-temperature acclimation and the subsequent cold stress on the malondialdehyde (MDA) levels have already been demonstrated in our earlier study (Szalai et al. 2018). Similarly, the patterns of the chlorophyll-*a* fluorescence parameters, including F_v_/F_m_, Y(II), Y(NPQ), and Y(NO) were similar to those we found in the previous experiment (data not shown). However, in the present work, we first demonstrate the effects of cold acclimation under different light conditions on a subsequent salt stress. The MDA measurements showed that while 50 mM salt only caused a slight, statistically not significant change in the lipid peroxidation, the effect of 150 mM NaCl was substantial, especially in the cold-acclimated plants, but regardless of the previously used light conditions (Fig. 2).

**Fig. 3:**
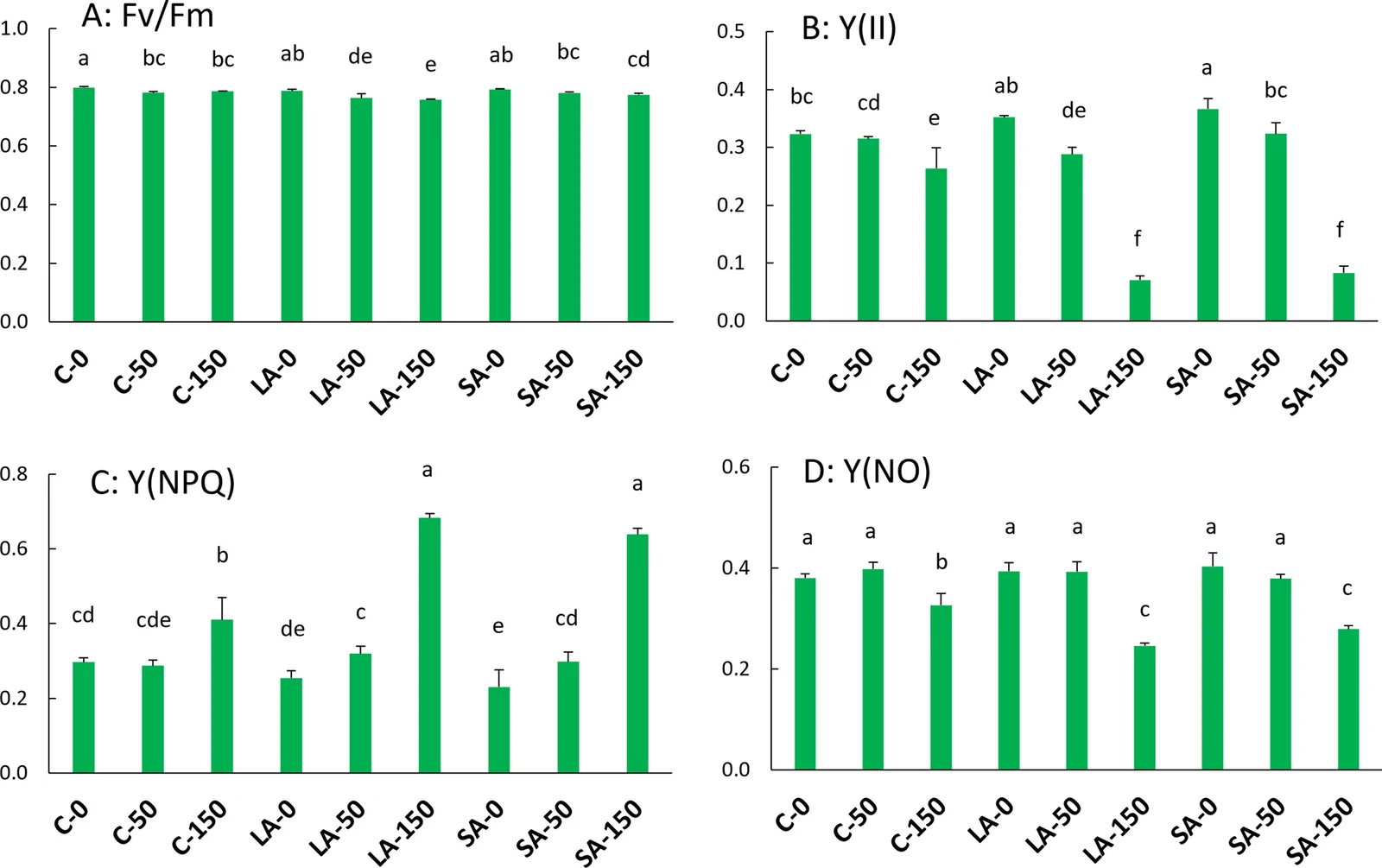
Changes in the chlorophyll-*a* fluorescence induction parameters (A: F_v_/F_m_, B: Y(II), C: Y(NPQ), D: Y(NO)) in the youngest fully expanded leaves of maize plants after exposure to 0, 50, or 150 mM NaCl with or without previous cold acclimation under different light conditions. *C* control, no cold acclimation; *LA* previously cold-acclimated at growth light, *SA* previously cold-acclimated in the shade. Mean and SD values, *n*=5. Different letters indicate significant differences at *p* < 0.05 level

In spite of the statistically significant differences, the F_v_/F_m_ parameter, representing the maximum quantum efficiency of Photosystem II, did not decrease substantially after salt treatments, indicating that neither the cold acclimation nor the high salinity caused very severe stresses (Fig. 3A). However, more pronounced effect was found in the light-adapted stage, where the Y(II) parameter, representing the light-adapted quantum yield, was significantly lower in the salt-treated plants, especially after 150 mM NaCl (Fig. 3B). This decrease was more intense in the previously cold-acclimated plants; however, the light intensity did not significantly affect the level of the decrease. The Y(NPQ) parameter, representing the regulated non-photochemical quenching, showed inverse changes, so it increased in plants exposed to 150 mM salt, especially in cold-treated plants (Fig. 3C). A similar trend (Y(II)) was also observed in Y(NO) (Fig. 3D). These results suggest that exposure of young maize plants to moderate low temperature may make the photosynthetic apparatus sensitive to a subsequent salt stress.

### Gene expression responses during cold acclimation and severe cold stress

**Fig. 4:**
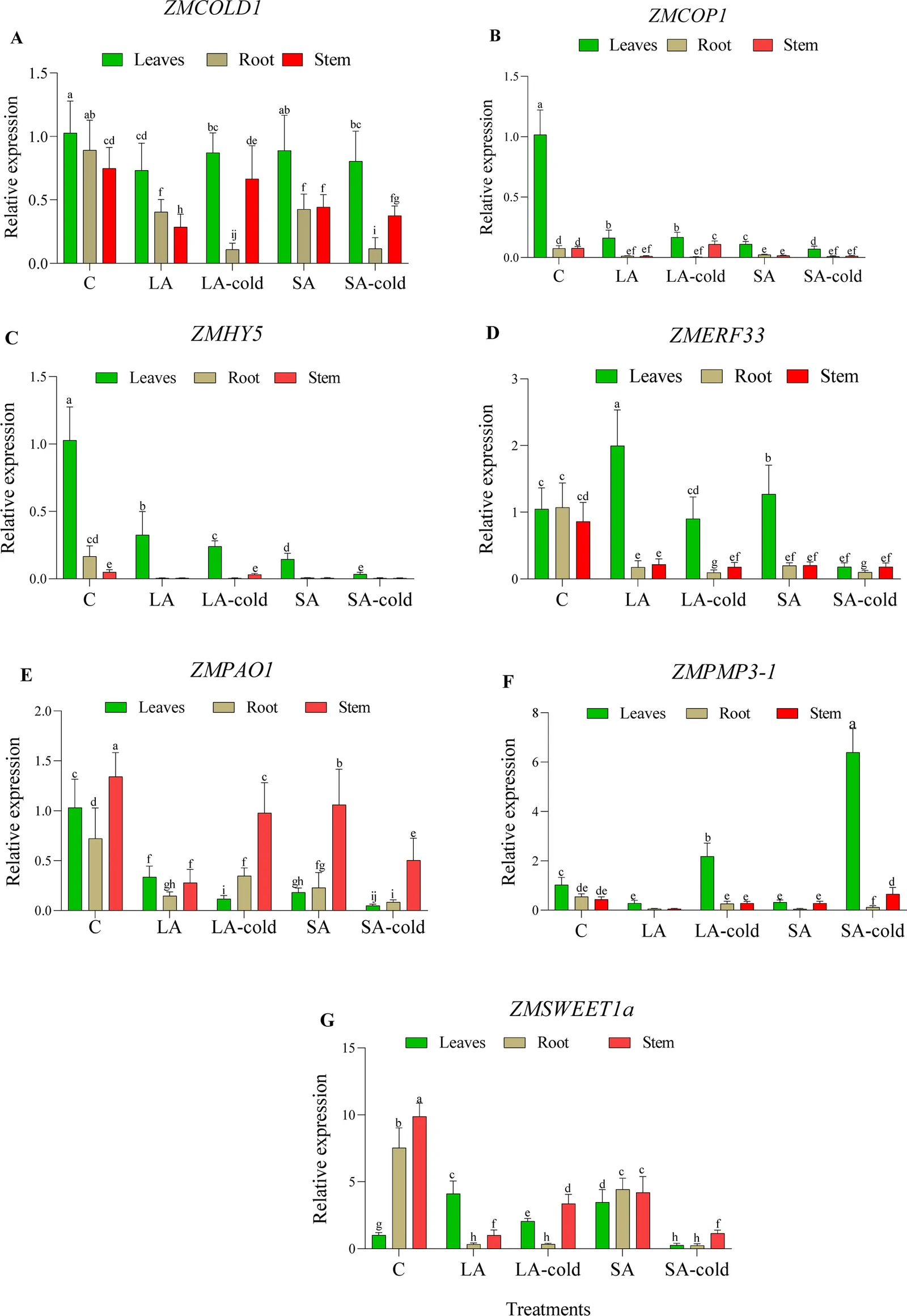
Relative expression levels of A) *ZmCOLD1*, B) *ZmCOP1*, C) *ZmHY5*, D) *ZmERF33*, E) *ZmPAO1*, F) *ZmPMP3-1*, and G) *ZmSWEET1a* in leaves, root, and stem of young maize plants under different temperature and light conditions. Control (C) represents plants at 22/20 °C before the cold acclimation period. LA and SA represent plants exposed to cold acclimation (at 15 °C) under normal light (LA, 250 µmol m^−2^ s^−1^) and shade conditions (SA, 50 µmol m^−2^ s^−1^). LA-cold and SA-cold represent light- and shade-acclimated plants after exposure to severe cold stress (5 °C) for 3 days. Bars represent means ± SD of three biological replicates, each with three technical replicates. Different lowercase letters indicate statistically significant differences among treatments (*P* < 0.05)

To investigate how cold acclimation under different light conditions modulates transcriptional stress responses, the expression of selected light, cold, osmotic and stress-related genes was analysed by RT–qPCR in leaves, stems and roots of young maize plants. Expression levels were examined during cold acclimation and subsequent severe cold exposure, and after salt stress applied following cold acclimation (Fig. 4).

During the cold acclimation period, the expression level of the *ZmCOLD1* gene was the highest in the leaves (Fig. 4A); however, low-temperature treatments did not cause significant changes in it. In contrast, low temperatures substantially reduced its expression levels in the stem, and especially in the roots. Interestingly, exposure to acclimating temperatures (15 °C) in the light led to a restoration after severe cold stress at 5 °C.

In contrast to *ZmCOLD1*, the *constitutively photomorphogenic1* (*ZMCOP1*) gene substantially decreased even at acclimating low temperatures in the leaves (Fig. 4B). In the roots and stems, its expression level was very low, compared to the leaves. A similar pattern was also observed in the case of *ELONGATED HYPOCOTYL5* (*ZmHY5*) gene (Fig. 4C). However, the expression level of the *ethylene response factor* (*ZmERF33*) gene increased after cold acclimation in the leaves, especially in the light, but then it decreased at 5 °C, especially after low-light cold acclimation conditions (Fig. 4D). Under control conditions, its expression levels in the roots and stem were similar to those of the leaves; however, in these organs, even the moderate cold led to a substantial reduction. The *polyamine oxidase ZMPAO1* gene was also sensitive to cold, although in the stem, under certain cold treatments, this decrease was less pronounced than in the leaves or in the roots (Fig. 4E). Plasma membrane protein 3 (PMP3) represents a class of small hydrophobic polypeptides with high sequence similarity. At low temperatures, the expression level of the *ZMPMP3-1* significantly decreased in all the organs tested; however, after exposure to severe stress, it substantially increased in the leaves, especially in plants, which were cold-acclimated in the shade (Fig. 4F). In contrast to the other genes tested in this study, the expression levels of the *ZmSWEET1a*, encoding a sugar transporter, were higher in the roots and stem than in the leaves of the control plants. However, in these organs, its expression was cold sensitive, not like in the leaves, where it increased at the acclimating temperatures (Fig. 4G).

Overall, three major transcriptional patterns emerged from the cold acclimation experiment. First, genes associated with light signalling (*ZmCOP1* and *ZmHY5*) showed predominantly leaf-specific expression and were strongly suppressed by low temperature. Second, several stress-responsive genes (*ZmERF33*, *ZmPMP3-1* and *ZmSWEET1a*) displayed pronounced organ-specific regulation depending on both temperature and light conditions. Third, although most genes were down-regulated by cold, *ZmPAO1* expression remained comparatively high in stems, suggesting that this organ maintained a distinct transcriptional response during cold acclimation.

### Gene expression responses to salt stress following cold acclimation

**Fig. 5:**
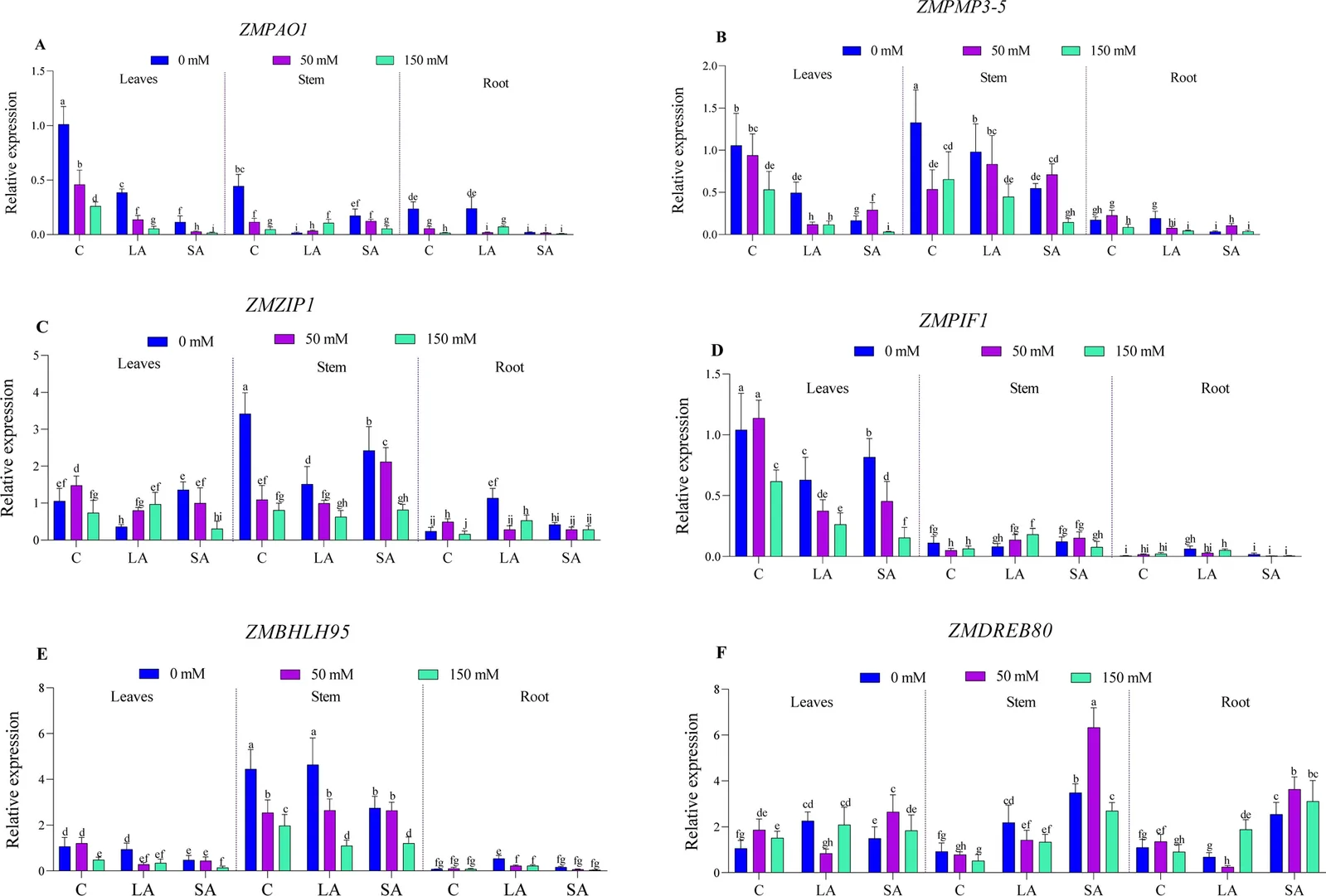
Relative expression of A) *ZmPAO1*, B) *ZmPMP3-5*, C) *ZmZIP1,* D) *ZmPIF1*, E) *ZmbHLH95*, and F) *ZmDREB80* in leaves, stems, and roots of maize plants exposed to salt stress (0, 50, and 150 mM NaCl) following cold acclimation under normal light or shade conditions. Control (C) represents plants at 22/20 °C before the cold acclimation period. LA and SA represent plants exposed to cold acclimation (at 15 °C) under normal light (LA, 250 µmol m^−2^ s^−1^) and shade conditions (SA, 50 µmol m^−2^ s^−1^). Bars represent means ± SD of three biological replicates, each measured with three technical replicates. Different lowercase letters indicate statistically significant differences among treatments (*P* < 0.05)

To assess whether cold acclimation under different light conditions alters transcriptional responses to salinity, gene expression was analysed after 3 days of salt treatment (0, 50 or 150 mM NaCl) at normal growth temperature (Fig. 5). Among the investigated organs, the expression level of the *ZmPAO1* gene was generally the highest in the leaves (Fig. 5A). After 3 days, the effect of cold acclimation was still obvious: it was lower in the cold-acclimated plants in all organs. Exposure to high salinity also reduced it in a concentration-dependent manner, especially in the shade-acclimated plants in the case of leaves and roots. Similar tendencies were also observed in the *ZmPMP3-5* gene, although its expression levels in the stem were slightly higher in the stem than in the leaves (Fig. 5B). The expression level of *Zn-related important gene iron-regulated transporter-like protein (ZmZIP1)* gene was also generally the highest in the stem, and both cold acclimation and salinity reduced them (Fig. 5C). In the leaves and in the stem, the most pronounced decrease in the cold-acclimated plants occurred in the LA plants, while in the root, cold acclimation in the light led to a slight increase in the expression of *ZmZIP1*. The *phytochrome-interacting factor ZmPIF1* expression levels were the highest in the leaves, followed by the stem and the root (Fig. 5D). Due to the low levels in the later organs, despite certain statistically significant differences, only the leaf values could be discussed reliably. Again, both cold acclimation and high salinity reduced its expression levels; however, substantial light-dependence could be observed. In contrast, the highest *BASIC HELIX–LOOP–HELIX TRANSCRIPTION FACTOR ZmBLBHL95* expression levels were detected in the stem (Fig. 5E). Cold acclimation reduced its level only in the shaded plants; however, salinity reduced it independently of the acclimation or light conditions. In the roots, although the values were low, a significant increase was detected in the LA plants. Among the investigated genes, the most remarkable cold acclimatory light-dependent changes could be detected in the expression levels of the *dehydration-responsive element-binding* (*ZmDREB80*) gene (Fig. 5F). In the control plants, their levels were similar in all the organs, and only slight changes, especially in the leaves, could be detected after salt exposure. However, cold acclimation also increased their expression, which were more pronounced in the LA plants in the leaves, and in the SA plants in the stem. In the root, this increase was only detectable in the SA plants. However, the most pronounced changes occurred in the plants exposed to moderate (50 mM) salt in the shade-acclimated plants, especially in the stem. At high salinity (150 mM), this increase was less pronounced. In the LA plants, such an increase could not be detected.

PCA further revealed a pronounced organ-specific pattern in the expression profiles of the investigated genes (Supplementary Figure S1). PC1 and PC2 explained 85.1% and 9.3% of the total variance, respectively, accounting for 94.4% of the overall variation. Stem samples showed the strongest separation and the greatest dispersion, whereas leaf and root samples largely overlapped, indicating more similar expression patterns between these two organs. The loading vectors also indicated a distinct separation of the control and salt-treated conditions, with the 50 and 150 mM NaCl treatments showing a broadly similar direction of variation.

Overall, following cold acclimation, salinity responses were likewise organ- and light-dependent. While most genes (*ZmPAO1*, *ZmPMP3-5*, *ZmZIP1* and *ZmPIF1*) exhibited an overall reduction in expression under increasing salinity, *ZmDREB80* showed the strongest interaction between previous light conditions and subsequent salt stress, particularly in stems, indicating that cold acclimation under different irradiance levels selectively reprogrammed transcriptional responses to salinity.

### Light-dependent metabolite changes at cold acclimation

In the next steps, detailed metabolomics analyses were carried out, focusing mainly on the primary metabolites, such as carbohydrates, amino acids, and organic acids; furthermore, certain plant growth regulators, phenylpropanoid-related compounds and polyamines. Untargeted analyses were also carried out, focusing on certain conjugated compounds.

**Fig. 6:**
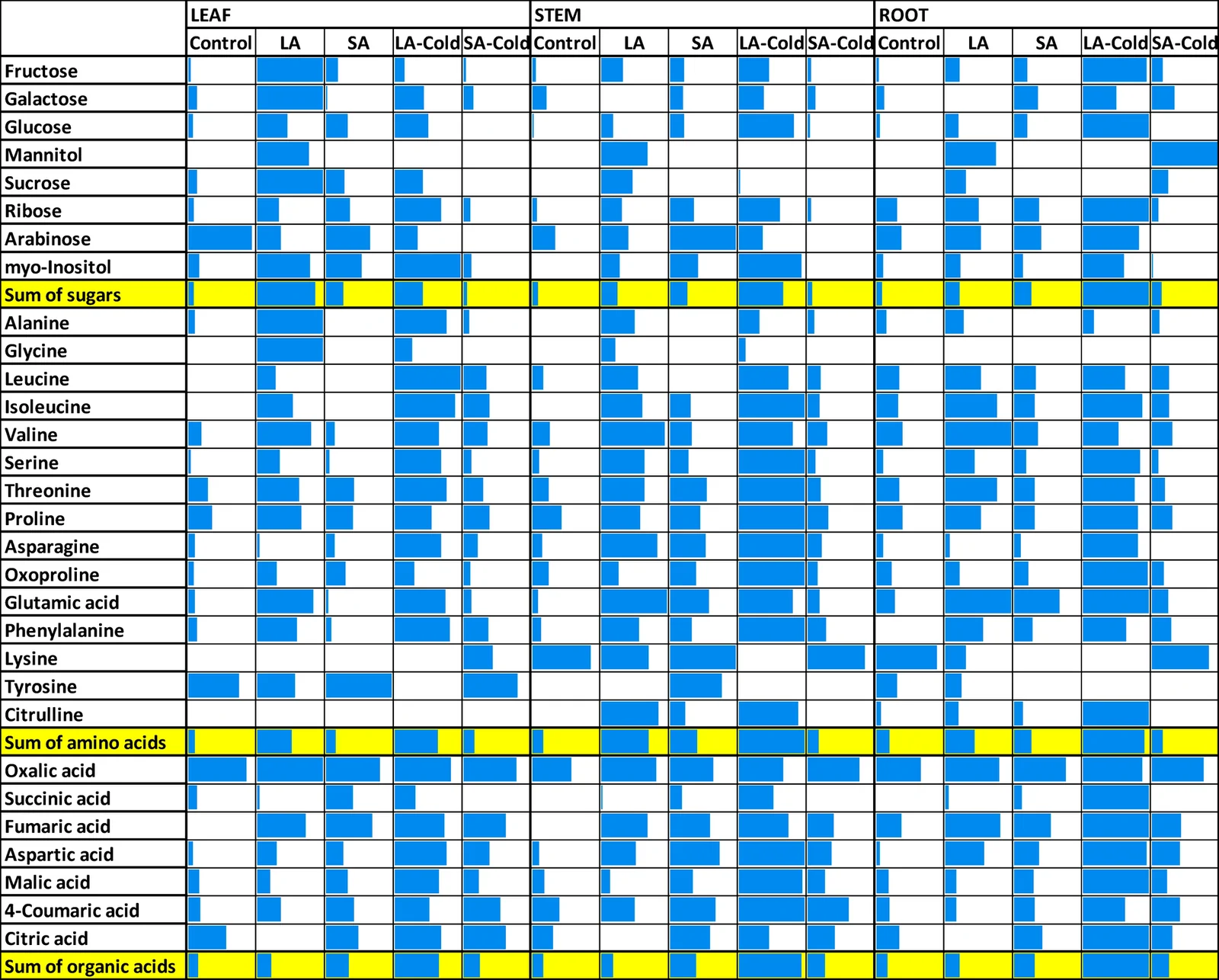
Relative abundance of selected primary metabolites in the leaves, stem, and roots of young maize plants after exposure to cold-acclimating temperatures in the light (LA) or under shade (SA) conditions and after exposure of the acclimated plants to severe cold stress at 5 °C (LA-cold and SA-cold). Lines with yellow background indicate the changes in sum of the given metabolite subgroups. The length of the bars indicates the relative amount of a given compound, considering all organs together. Absolute mean and SD values together with the results of the statistical analysis can be seen in Supplementary Table S4: “Primary metabolites cold”

First, the primary metabolites were determined in the leaf, stem and root of control and cold-acclimated (LA and SA) plants using the GCxGC–TOF technique (Fig. 6; Supplementary Table S4). In the case of sugar or sugar alcohol compounds, the effect of light is detectable in all three organs. As it was expected, usually, the most pronounced increase occurred mainly in the leaves after exposure to acclimating temperatures in the light. Interestingly, after extreme cold stress (LA-Cold and SA-Cold), in most cases, the values ​​(except for glucose, ribose and myo-inositol) did not increase but rather decreased in light-acclimated leaves, but did in the shoot or root. In the shade cold-acclimated plants, these changes were much less pronounced. The behaviour of arabinose, a cell wall compound, was different: the cold-induced growth was not detected in the leaves, but it increased in the stem of the SA plants.

Similarly to the sugars, cold acclimation in the light significantly increased the levels of most of the amino acids in the leaves, while this increase was not significant under low light conditions (Fig. 6; Supplementary Table S4). We made a similar observation in the case of roots. In contrast to these, a substantial increase in the stem also occurred in the SA-cold plants. Except for the tyrosine and citrulline in the leaves, at severe stress, the high amino acid levels were kept or even further increased in the LA-cold plants, but not in the SA-cold acclimated ones. Nevertheless, results clearly showed that for root amino acid levels to increase in the cold, acclimation must occur in light. In contrast to sugars and amino acids, in the case of organic acids, the role of light during the acclimation period is less decisive, but in severe cold, it is detectable for the key compounds of the tricarboxylic acid (TCA) cycle, such as succinate, fumarate, malate, and citrate (Fig. 6; Supplementary Table S4).

**Fig. 7:**
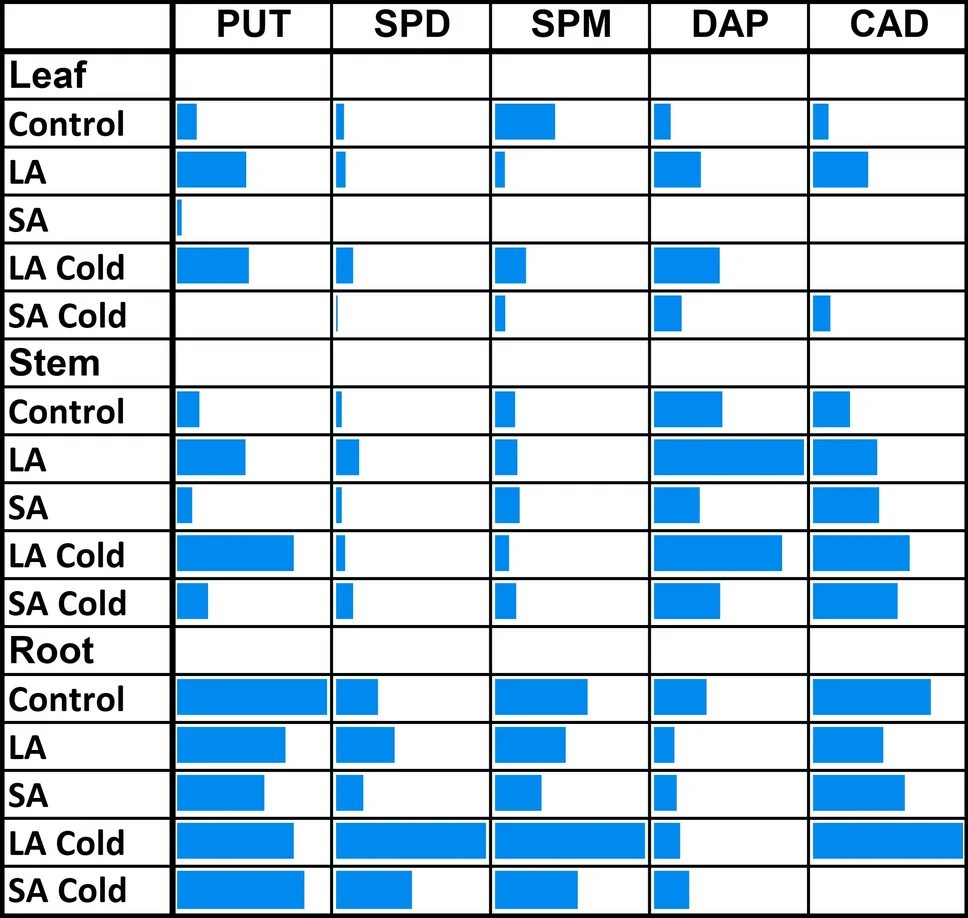
Relative abundance of polyamines in control, and in light and shade cold acclimation plants before (LA and SA) and 3 days after severe cold stress (LA-cold and SA-cold) at 5 °C. The length of the bars indicates the relative amount of a given compound, considering all organs together. Absolute mean and SD values together with the results of the statistical analysis can be seen in Supplementary Table S4: “Polyamine cold”

In the examined organs, PUT was the most abundant polyamine, and interestingly, DAP was also present in significant amounts (Supplementary Table S4). In comparison, SPD, SPM, and CAD, which latter is synthesized in a separate pathway, were present in only trace amounts. The effect of cold treatments was substantial for PUT and, to a lesser extent, for DAP, and mainly in the leaves and stem. However, the cold-induced increase was only significant under LA conditions (Fig. 7). In contrast, a significant increase in the SPD and SPM levels occurred under severe cold stress in the roots of LA plants.

**Fig. 8:**
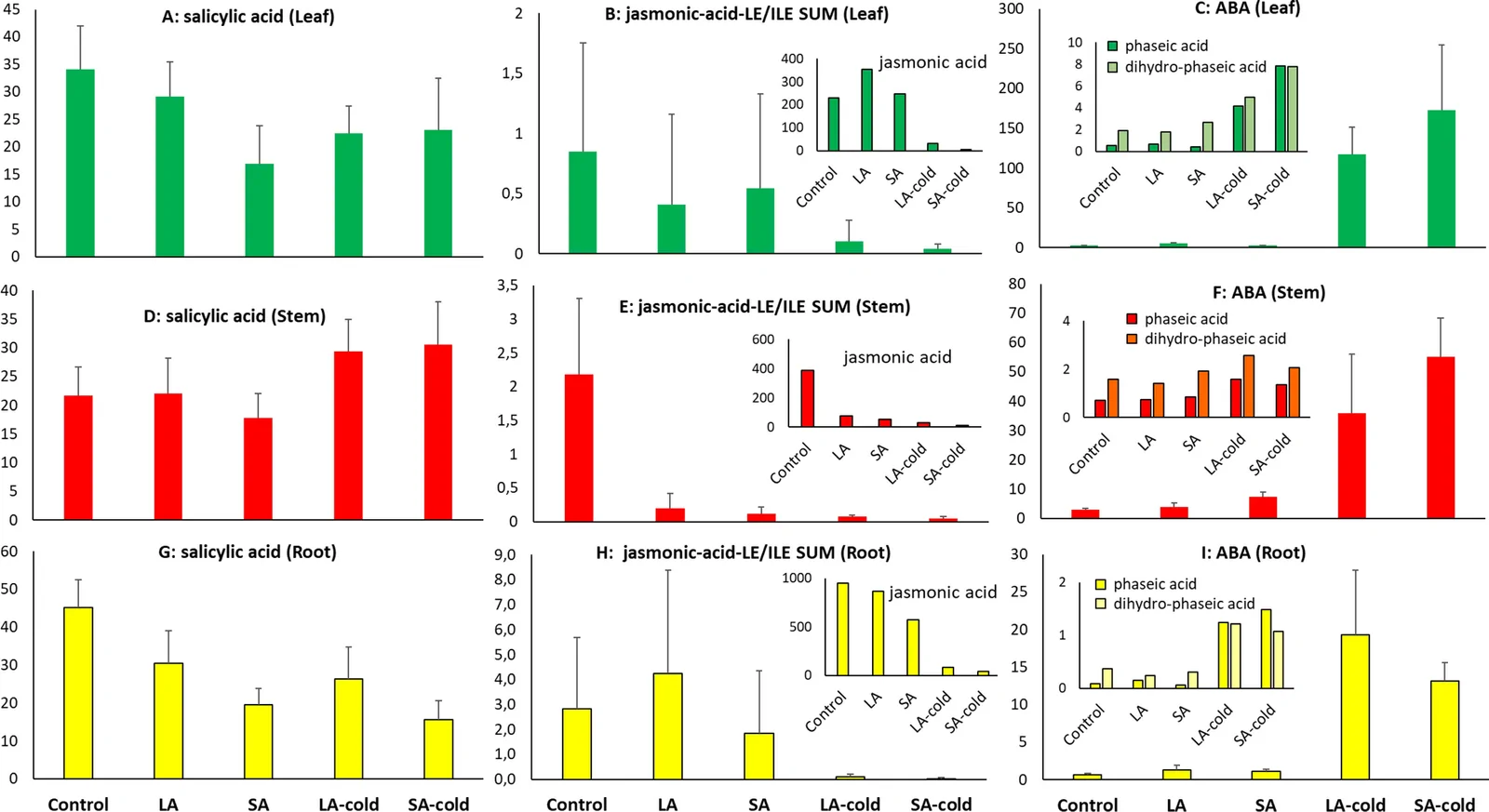
Changes in the contents of certain plant hormones, such as salicylic acid (**A, D, G**), jasmonic acid–Leu/Ile conjugates with the free jasmonic acid values in the inserts (**B, E, H**), and abscisic acid (ABA) with its degradation products phaseic acid and dihydro-phaseic acid (light colours) in the inserts (**C, F, I**) in the leaves (green bars, **A, B, C**) stem (red bars, **D, E, F**), and roots (yellow bars, **G, H, I**) of the control, and light and shade cold acclimation plants before (LA and SA) and 3 days after severe cold stress (LA-cold and SA-cold) at 5 °C. Data are given in ng g^−1^ FW. Absolute mean and SD values together with the results of the statistical analysis can be seen in Supplementary Table S4: “Hormones cold”

Among the plant hormones determined using our method, salicylic acid was detectable in similar amounts in all three organs (Fig. 8; Supplementary Table S4). We obtained a similar pattern in the leaf and root: it was present in smaller amounts in both acclimation and severe cold than in the control, and this was mainly evident at low light intensity. In contrast, in the stem it did not change during the acclimation period, but it increased in severe cold, irrespective from the previous light conditions. This may also suggest that it could have been formed in the leaves and root independently of the similar pattern, and not by transport through the stem. The active form of jasmonic acid, the Leu/Ile conjugate, did not change statistically in the leaf and root at cold acclimation, but it decreased significantly in the stem (Fig. 8; Supplementary Table S4). On the other hand, a significant decrease was observed in all organs under severe cold. The amount of free jasmonic acid changed similarly to the active form. In contrast to these, ABA increased slightly under acclimating and greatly under severe cold conditions (Fig. 8; Supplementary Table S4). This increase was mainly expressed in leaves and stems of light-acclimated plants, and mainly in roots of dark-acclimated plants. The change was also followed by the amount of its degradation product, so the increase did not result from a decrease in degradation, but from an increase in biosynthesis.

**Fig. 9:**
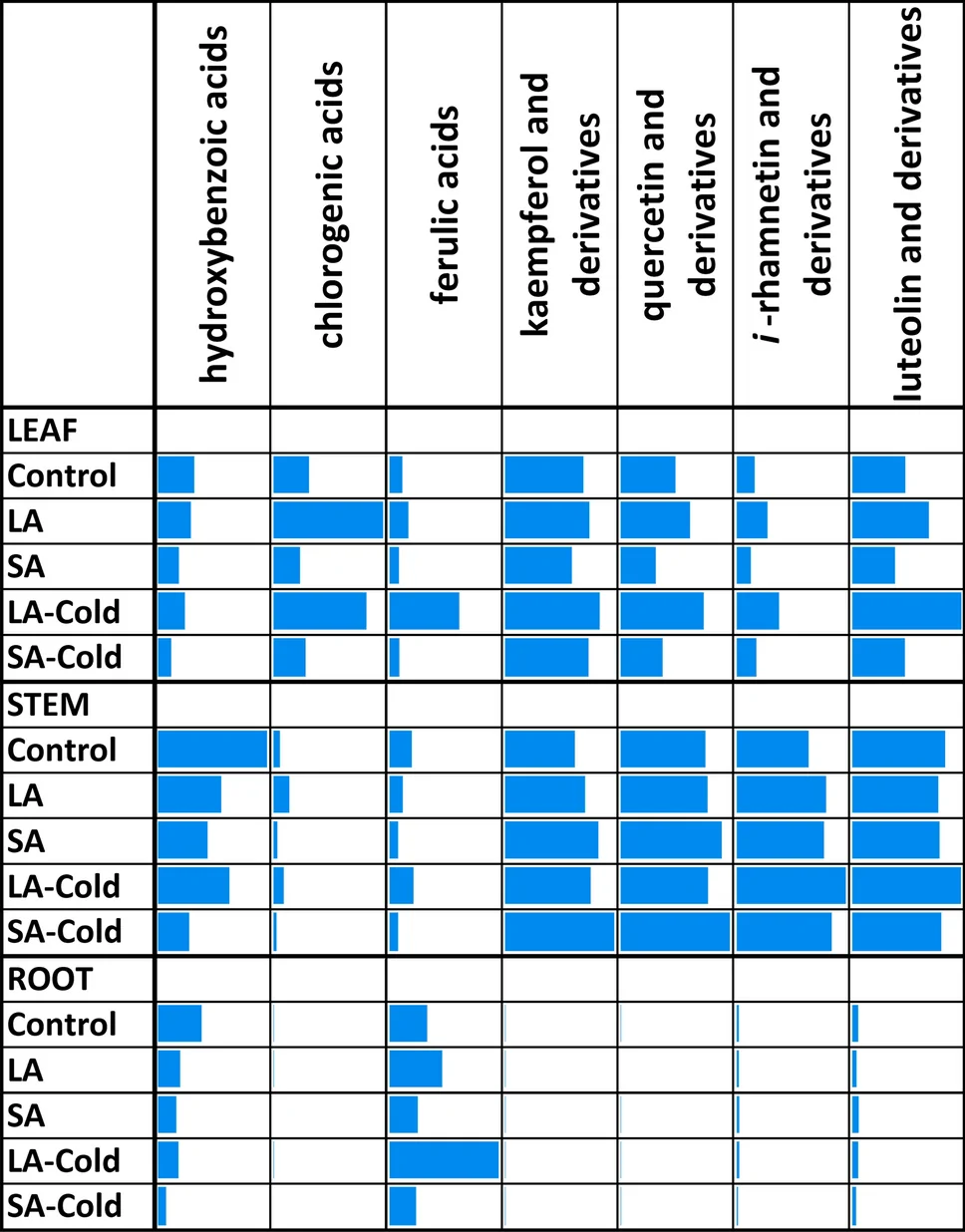
Changes in the contents of the sum of certain phenolics groups in the control, and light and shade cold-acclimation plants before (LA and SA) and 3 days after severe cold stress (LA-cold and SA-cold) at 5 °C. The length of the bars indicates the relative amount of a given compound, considering all organs together. Absolute mean and SD values together with the results of the statistical analysis for the individual compounds can be seen in Supplementary Table S4: “Phenolics cold”

Certain elements of the phenylpropanoid biosynthesis under low temperature conditions were also investigated (Fig. 9; Supplementary Table S4). The hydroxybenzoic acids were mainly detected in the stem, but the cold and light effects are not obvious. The amounts of ferulic acids were the highest in the root, except for one of the isomers, which is a lot in the leaf, but its absolute value is relatively low, compared to the other isomers. Ferulic acids showed a substantial increase in the leaf and root when exposed to severe cold, when they were previously acclimated in the light. Chlorogenic acids are present mainly in the leaves, and they increase at low temperatures when also exposed to light. Flavonols (kaempferol, quercetin, *i*-rhamnetin and their derivatives) and flavones (luteolin and its derivatives) accumulated mainly in the leaves and stem. Changes, detected in the kaempferol levels, are not substantial. Quercetins, *i*-rhamnetins and luteolins have similar patterns in the leaves; an increased amount can be seen in the leaves of light-acclimated plants either at acclimation or low temperature. A slight increase could be seen in the level of *i*-rhamnetins and luteolins in the stem of LA plants at 5 °C, while no changes are observed in the case of quercetin.

**Fig. 10:**
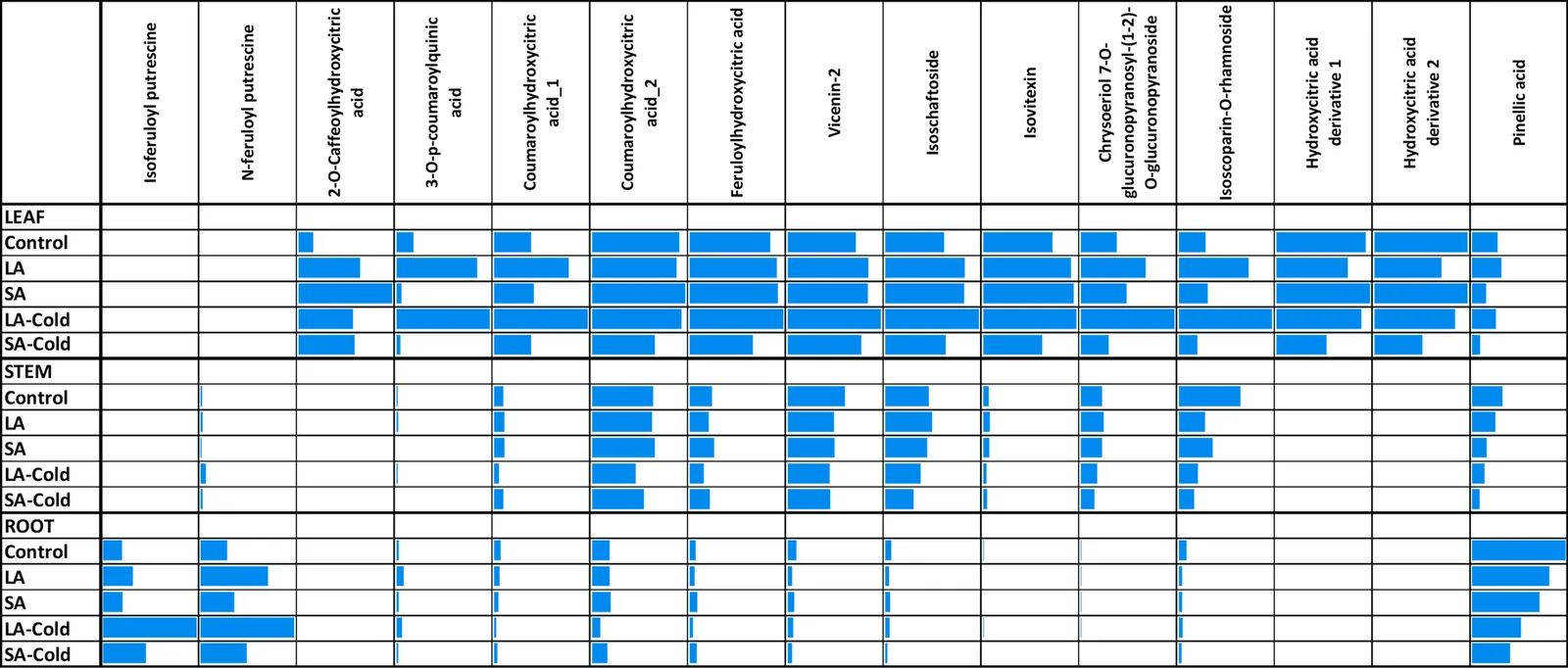
Changes in certain conjugated compounds in control, and in light or shade cold-acclimation plants before (LA and SA) and 3 days after severe cold stress (LA-cold and SA-cold) at 5 °C. The length of the bars indicates the relative amount of a given compound, considering all organs together. Absolute mean and SD values together with the results of the statistical analysis can be seen in Supplementary Table S4: “Untargeted cold”

Using the LC/MS/TOF technique, further stress-related compounds or their derivatives could also be detected (Fig. 10; Supplementary Table S4). While the changes in the PUT in the roots during the cold treatments were not substantial, its isoferuloyl and N-feruloyl derivatives significantly increased after severe cold stress, but mainly in the LA, and to a much lesser extent in the SA plants. These derivatives could not be, or in a few cases, only in trace amounts, detected in the leaves or stem. Besides the above-mentioned PUT derivatives, pinellic acid was the other compound, which was in substantially higher amount in the root than in the leaves or stem. However, its amount showed a significant decrease with the decreasing temperature, especially in the SA plants. Although most of the investigated compounds could also be detected in the stem, and in certain cases, they also showed statistically significant cold responses, majority of them were in the highest amounts in the leaves (Fig. 10; Supplementary Table S4).

The detected compounds included 3 coumaroyl derivatives, and two of them, 3-O-p-coumaroylquinic acid and coumaroylhydroxycitric acid_1 but not the coumaroylhydroxycitric acid_2 showed the most pronounced light-dependent increase after exposure to low temperatures. Similarly, high level of light-dependency was detected in the changes in the isoscoparin-O-rhamnoside contents during the cold treatments.

Overall, the results support the view that the metabolic background of cold adaptation is strongly light-dependent and results in organ-specific response patterns. Changes in primary metabolites, polyamines, hormones, and phenylpropanoid derivatives collectively suggest that light acclimation is essential for efficient cold tolerance, especially in leaves and roots. The distinct metabolic rearrangements observed under severe cold stress reflect a complex, integrated regulation of biosynthetic and signalling processes.

### Metabolomic changes after salt treatments in the cold-acclimated plants

**Fig. 11:**
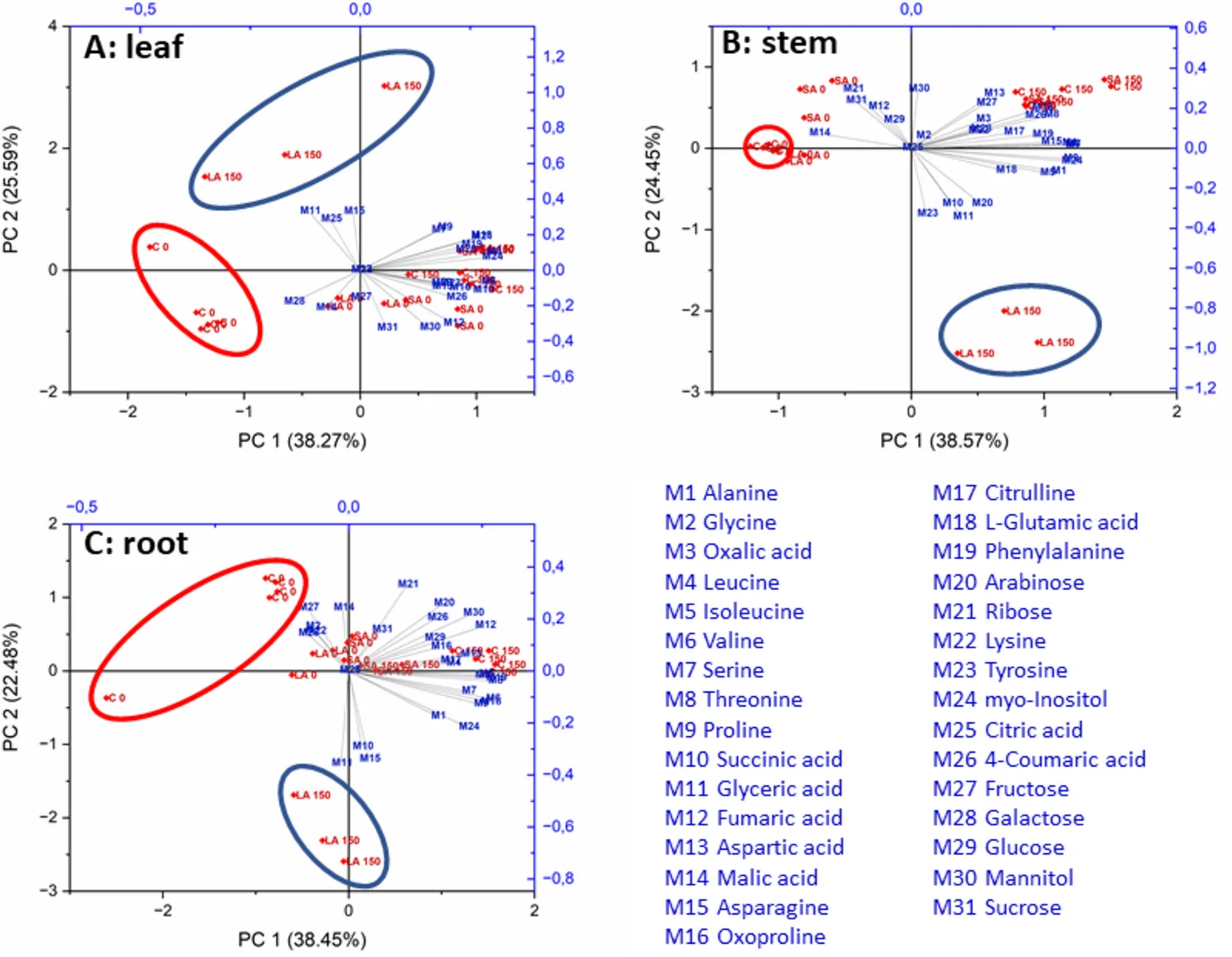
Principal component analysis (PCA) of primary metabolites in the leaves (A), stems (B), and roots (C) of young maize plants exposed to 0, 50, or 150 mM NaCl following different cold-acclimation treatments. C indicates plants without previous cold acclimation; LA indicates plants previously cold-acclimated under growth-light conditions; and SA indicates plants previously cold-acclimated under shade conditions. The numbers following C, LA, or SA indicate the NaCl concentration (0, 50, or 150 mM). Red circles indicate the C treatment groups, whereas blue circles indicate the LA 150 group. M1–M31 indicate the individual primary metabolites included in the PCA; metabolite identities are given in the list next to the plots

To see whether cold treatment under different light conditions affected the salt stress responses of the tested maize plants at metabolite level, we subjected certain groups of compounds to PCA analysis. Here, we mainly focused on the effects of treatment with 150 mM salt on different organs, and the results of PCA analyses on the compounds of primary metabolites—as compounds generally playing role basic biochemical processes—are presented in Fig. 11. For all three organs, the first two principal components explained a substantial proportion of the total variance, allowing the major differences among the treatment groups to be visualized in the two-dimensional PCA plots. The PCA plots show that C-0 plants, particularly in the leaves and roots, and LA-150 plants in all three organs were clearly separated from the other treatment groups (SA-0, C-150 and SA-150). In the stem, SA-0 was also separated from the other groups, whereas C-0 and LA-0 were positioned relatively close to each other (Fig. 11B). In general, C-150 and SA-150 were positioned relatively close to each other in all three organs, whereas LA-150 was clearly separated from these groups. This suggests that the light conditions during cold acclimation influenced the subsequent metabolic response to salinity, particularly in the LA plants. In the leaf samples, the major separation of the treatment groups occurred along PC2, with C-0 and LA-150 showing distinct positions from the other groups (Fig. 11A). In the stem, LA-150 was clearly separated from the other treatment groups, mainly along PC2, while the separation of SA-0 from the salt-treated groups was primarily associated with PC1 (Fig. 11B). Thus, both PC1 and PC2 contributed to the separation of the treatment groups in the stem, with PC2 particularly distinguishing the LA-150 plants from the other groups. This may also support that the stem not only behaves as a passive transport organ, but also undergoes active metabolic reprogramming as a result of the treatment, which strengthens the systemic nature of the plant response. In the roots, as in the leaves, the major separation of the treatment groups occurred along PC2, with C-0 and LA-150 clearly separated from the other groups (Fig. 11C). In general, Fig. 11, therefore, shows that in the level of the primary metabolites, the treatment does not induce isolated metabolite changes in the plant, but rather a coordinated, pathway-level metabolic reorganization, which manifests itself differently in each organ, but indicates a response system that extends throughout the plant. Based on the PCA loading patterns, the separation of treatment groups was associated with changes in TCA-cycle intermediates (citric acid, malic acid, succinic acid, and fumaric acid) and soluble sugars (glucose, fructose, and sucrose), pointing to changes in central carbon metabolism. Metabolites related to nitrogen metabolism, including glutamic acid, aspartic acid, asparagine, and citrulline, also contributed to the separation, suggesting changes in carbon–nitrogen metabolism. In the leaves, compounds related to photorespiration and redox regulation (glycine, serine) dominate; in the stem, precursors of carbon–nitrogen redistribution and the phenylpropanoid pathway (phenylalanine, 4-coumaric acid) contribute to the separation, while in the root, metabolites related to N storage and respiration (asparagine, succinic acid) are decisive. Osmoprotectant-related metabolites, including proline, mannitol, and myo-inositol, also contributed to the observed metabolic differences across the three organs, consistent with an osmotic adjustment component of the response. Overall, an organ-specific but coordinated re-tuning of carbon, nitrogen and redox metabolism underlies the different metabolic responses of the treated plants.

**Fig. 12:**
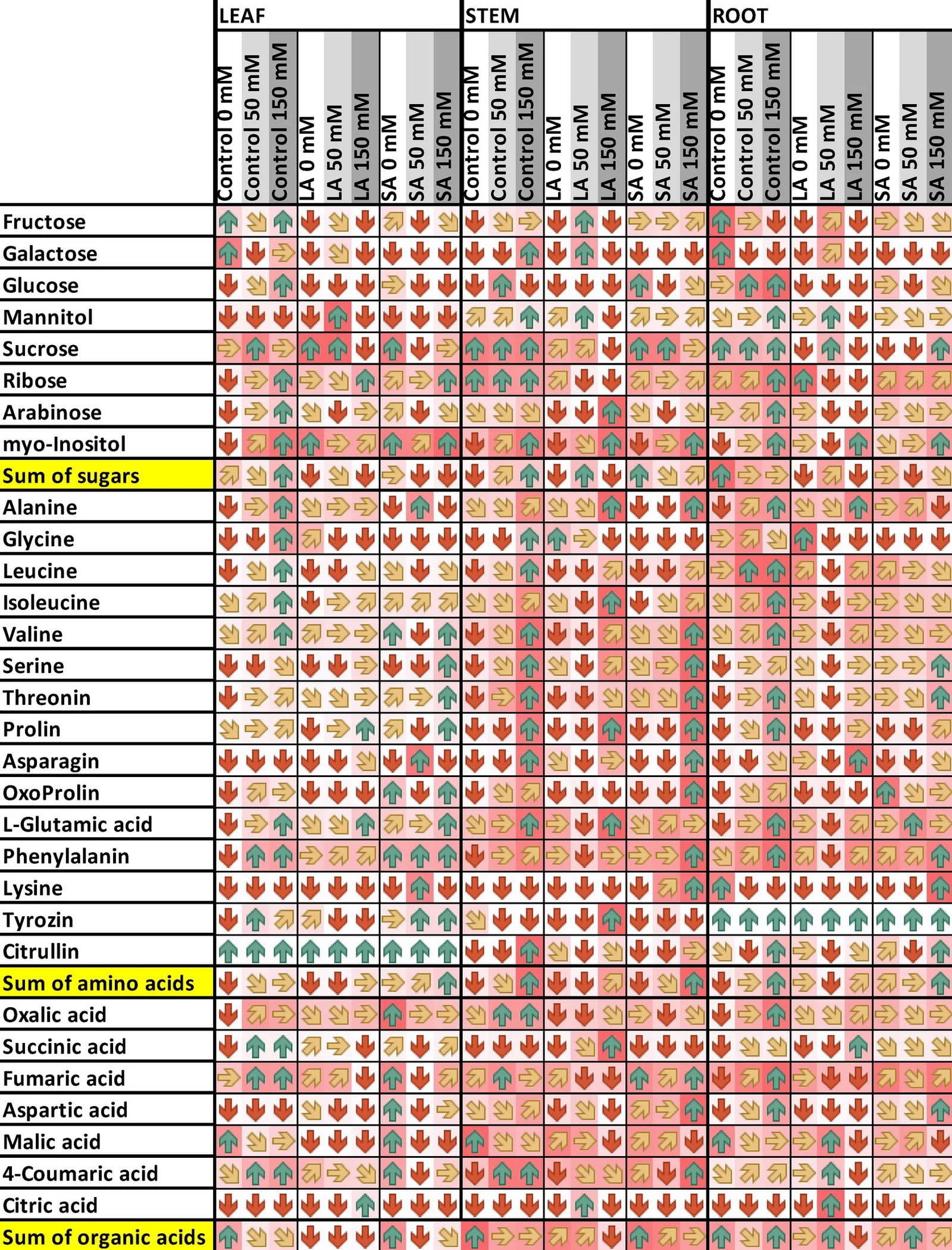
Heatmap of relative abundance of selected primary metabolites in the leaves, stem, and roots of young maize plants exposed to 0, 50, or 150 mM NaCl with or without previous cold acclimation under different light conditions. Lines with yellow background indicates the changes in sum of the given metabolite subgroups. Darker colours indicate higher concentrations. Arrows indicate the relative changes within each organ. Absolute mean and SD values together with the results of the statistical analysis can be seen in Supplementary Table S4 “Primary metabolites salt”. (*LA* light acclimated, *SA* shade acclimated, *0, 50 and* *150 mM* NaCl concentration)

When the individual compounds were investigated in the salt-treated plants, it was found that in the case of sugars, the effects of the previous cold acclimation and light might also appear in all three organs (Fig. 12; Supplementary Table S4). The effects of high salinity (150 mM) in the control, non-acclimated leaves were obvious: there was a general increase in the sugar levels. However, in the cold-acclimated plants, this increase was not significant. The increasing salt concentration also led to high amino acid concentrations in all the investigated organs. This increase was least pronounced in the light-acclimated plants (Fig. 12; Supplementary Table S4). The role of light is less decisive for organic acids, but there was a difference for certain acids (e.g., succinic acid) (Fig. 12; Supplementary Table S4).

**Fig. 13:**
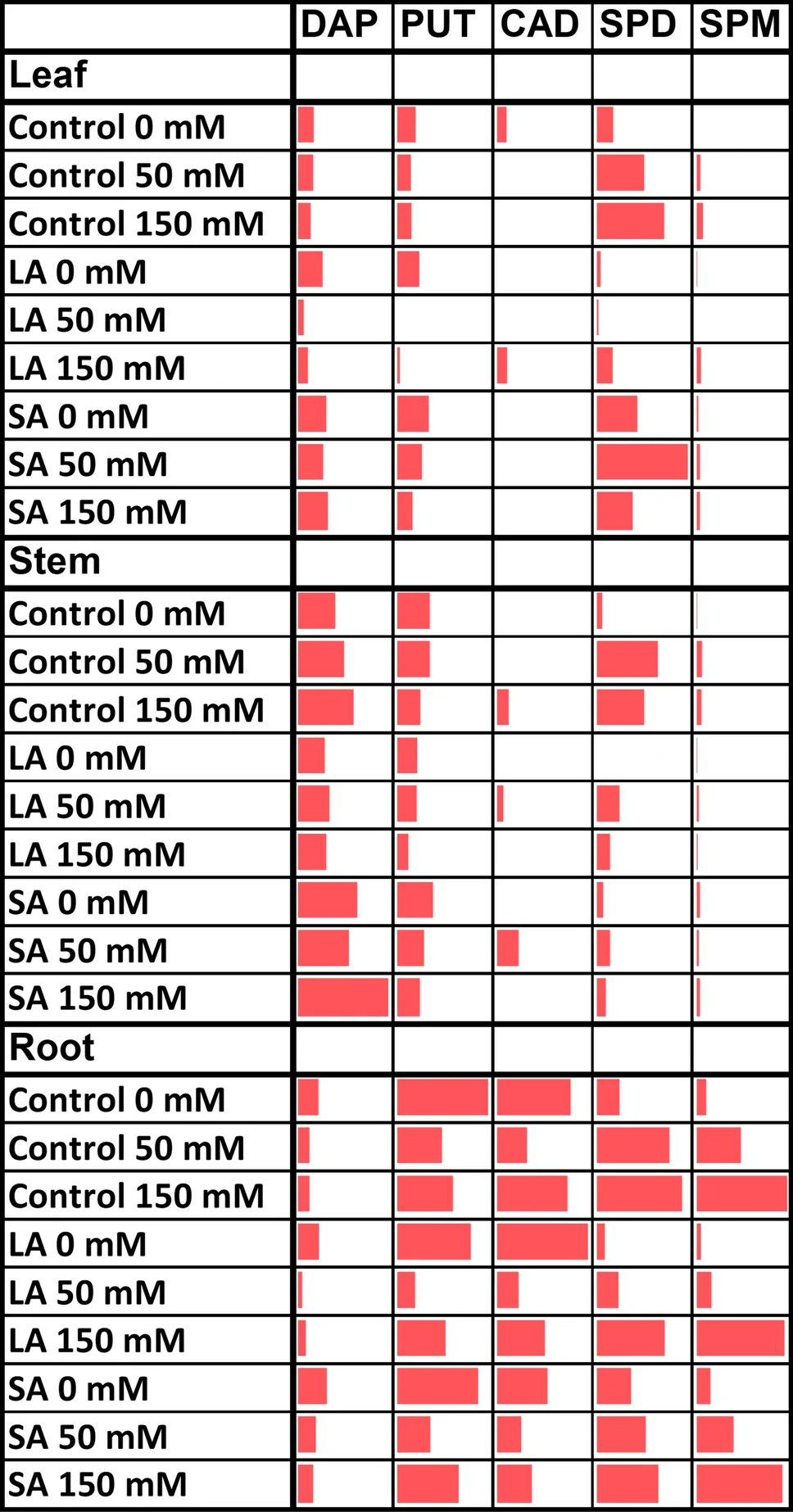
Relative abundance of polyamines in the leaves, stem, and roots of young maize plants exposed to 0, 50, or 150 mM NaCl with or without previous cold acclimation under different light conditions. The length of the bars indicates the relative amount of a given compound, considering all organs together. Absolute mean and SD values together with the results of the statistical analysis can be seen in Supplementary Table S4: “Polyamine salt”. (*LA* light-acclimated, *SA* shade-acclimated, *0, 50 and 150 mM* NaCl concentration)

Among the polyamines, increasing salinity caused the most remarkable changes in the spermidine levels (Fig. 13; Supplementary Table S4). Interestingly, in the LA plants this increase was substantially down-regulated. Interestingly, substantial catabolic activities could be observed in the stem, as represented by the high level of the DAP values. However, this was reduced in plants, which were previously cold-acclimated in the light. Furthermore, intensive SPD and SPM synthesis was detected in the roots under saline conditions, but not in the leaves or stem (Fig. 13; Supplementary Table S4).

**Fig. 14:**
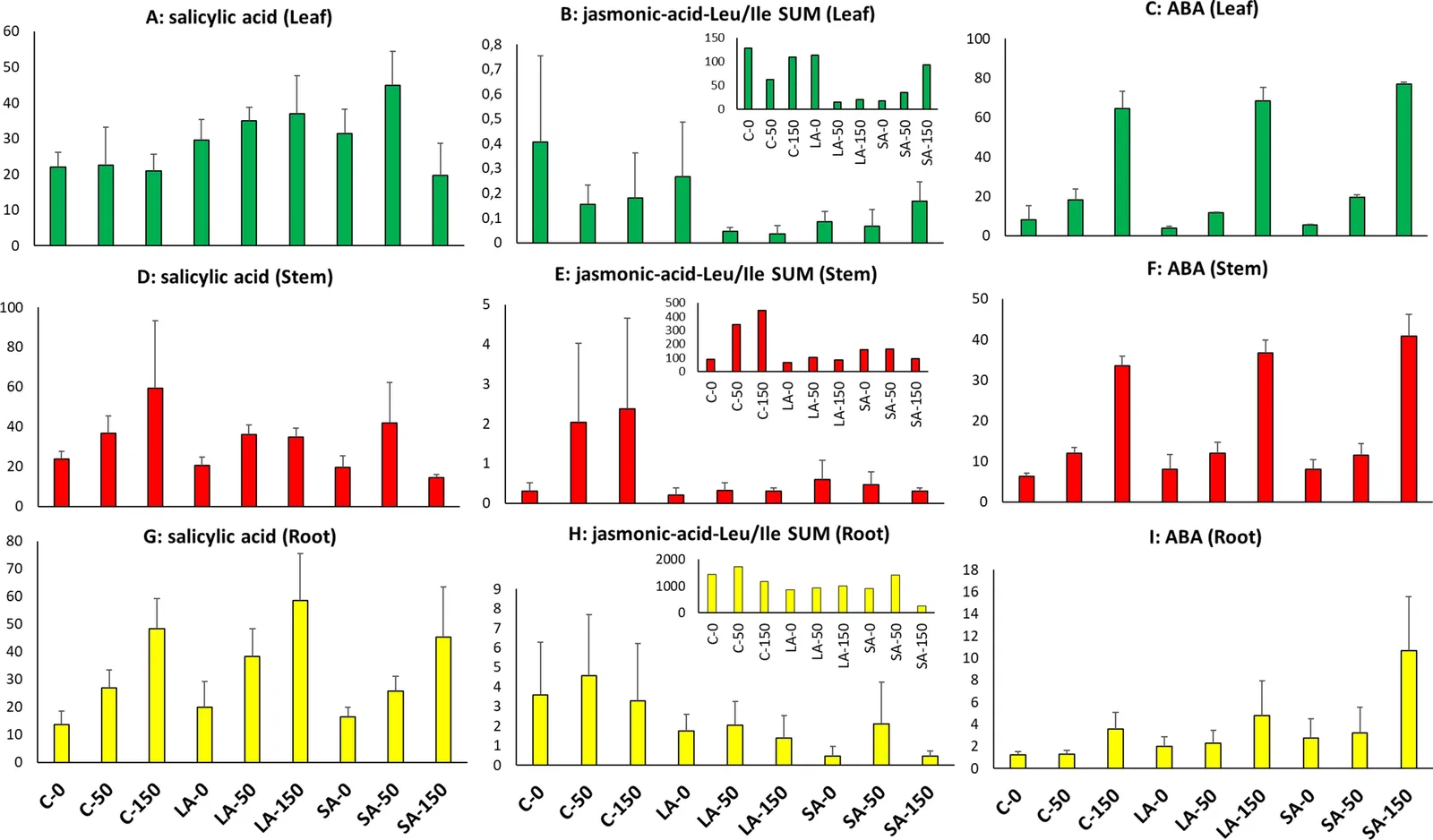
Changes in the contents of plant hormones, such as salicylic acid (**A, D, G**), jasmonic acid–Leu/Ile conjugates (**B, E, H**) with the free jasmonic acid values (in the inserts), and abscisic acid (ABA; **C, F, I**) in the leaves (green bars; **A, B, C**) stem (red bars; **D, E, F**), and roots (yellow bars; **G, H, I**) of young maize plants exposed to 0, 50, or 150 mM NaCl with or without previous cold acclimation under different light conditions. Data are given in ng g^−1^ FW. Absolute mean and SD values together with the results of the statistical analysis can be seen in Supplementary Table S4: “Hormones salt”. (*LA* light-acclimated, *SA* shade-acclimated, *0, 50 and 150 mM* NaCl concentration)

Among the plant hormones, abscisic acid, jasmonic acid and isoleucine/leucine conjugate, and salicylic acid were detected in all three examined organs (Fig. 14; Supplementary Table S4). Abscisic acid showed the greatest change in all organs upon salt treatment: a substantial increase was detected in salt concentration-dependent manner. This change also showed a similar pattern in its degradation product, indicating that the increase is primarily due to the enhancement of abscisic acid synthesis. A similar salt concentration-dependent change was also detected in the salicylic acid content of the root. In the case of the other organs and hormones, the changes were either not significant or were influenced by other factors. In the case of control plants, the applied salt treatments did not cause any change in the salicylic acid content, but in the pre-cold-treated plants, a small increase was observed in some cases as a result of salt treatment. In contrast, in the stem of the control plants, the salicylic acid content increased depending on the salt concentration, while in the cold-treated plants this change was not consistent. Similarly, a substantial increase was also detected in the amounts of jasmonic acid and jasmonic acid Leu/Ile conjugates in the stems of the control plants, but in the pre-cold-treated plants, the amounts of these compounds were much lower, so the differences were not detectable either. This was observed not only in the stem, but also in the leaves and, to a lesser extent, in the root (Fig. 14; Supplementary Table S4).

**Fig. 15:**
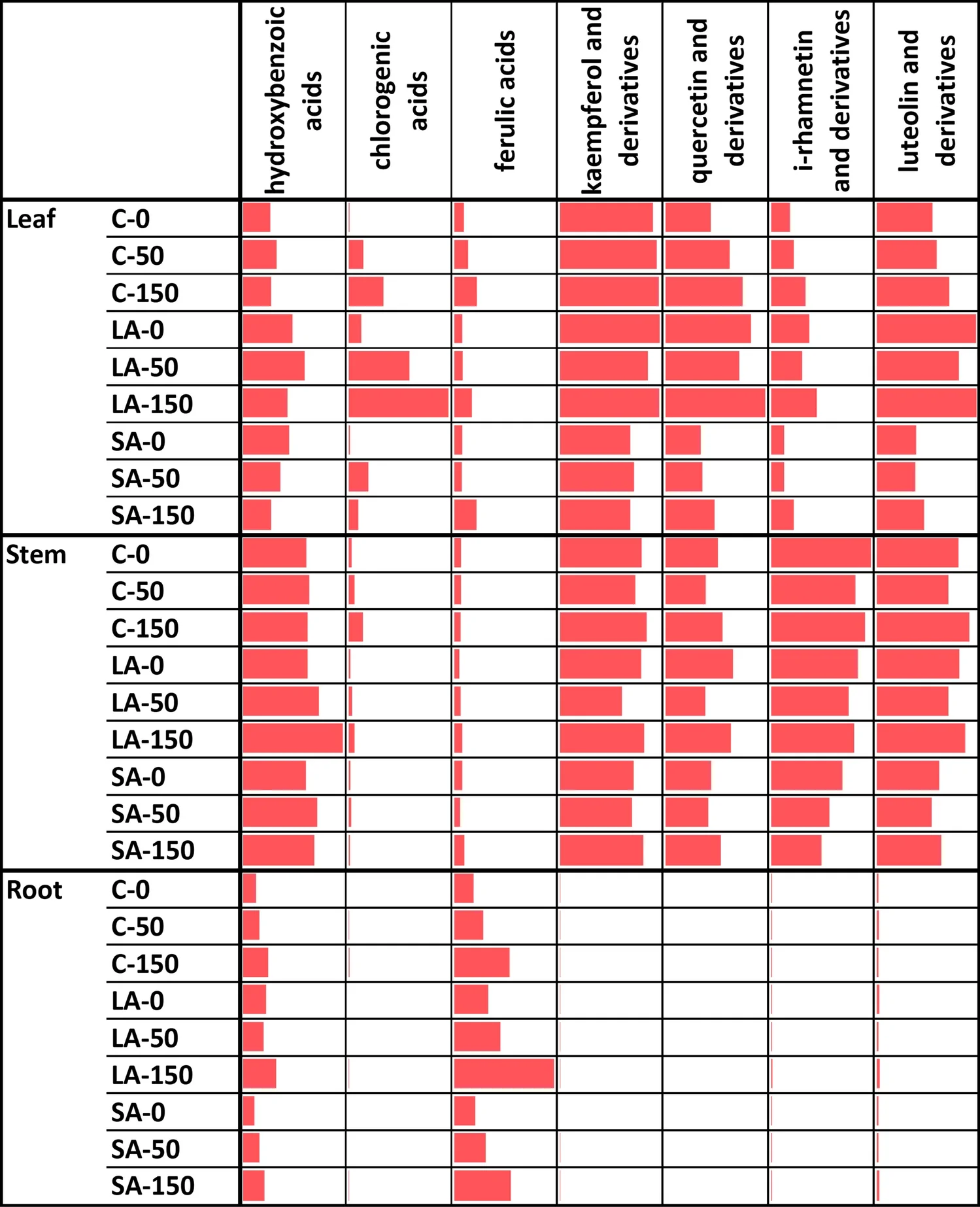
Changes in the contents of the sum of certain phenolics groups in the leaves, stem, and roots of young maize plants exposed to 0, 50, or 150 mM NaCl with or without previous cold acclimation under different light conditions. The length of the bars indicates the relative amount of a given compound, considering all organs together. Absolute mean and SD values together with the results of the statistical analysis can be seen in Supplementary Table S4: “Phenolics salt”. (*LA* light-acclimated, *SA* shade-acclimated, *0, 50 and 150 mM* NaCl concentration)

Among the phenylpropanoid compounds (Fig. 15; Supplementary Table S4), the effects of salt or cold treatments on the hydroxy acid contents, including hydroxybenzoic acids, chlorogenic acids and ferulic acids, are not generally obvious; however, in certain cases these effects are significant. The amount of chlorogenic acids increased in the leaves of salt-treated plants in a concentration-dependent manner (Fig. 15). The effect of light was also detected, as this increase was more pronounced in light-acclimated plants. Furthermore, in the case of ferulic acids, a significant increase could be detected at 150 mM salt; and the effect of light is also visible: the increase is more pronounced in the roots of light-acclimated plants. The luteolin was only detectable in the leaf and stem, and the effect of light could also be detected. Similarly, the quercetin content increased less in the dark, cold-acclimated plants. In the case of *i*-rhamnetin the effect of salt is not significant, but the effect of cold acclimation is visible: slightly lower values were detected in SA plants (Fig. 15).

**Fig. 16:**
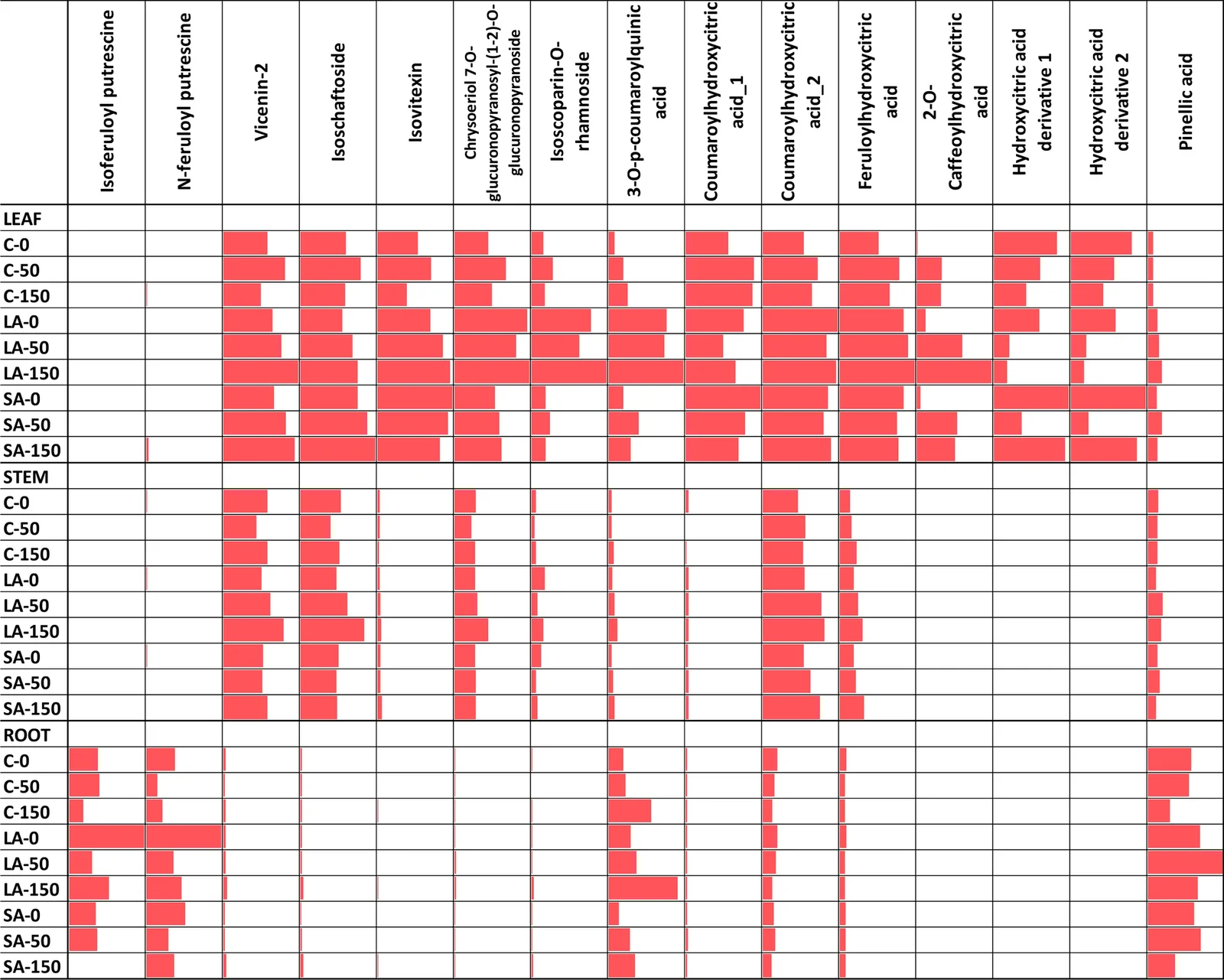
Changes in certain conjugated compounds in the leaves, stem, and roots of young maize plants exposed to 0, 50, or 150 mM NaCl with or without previous cold acclimation under different light conditions. The length of the bars indicates the relative amount of a given compound, considering all organs together. Absolute mean and SD values together with the results of the statistical analysis can be seen in Supplementary Table S4: “Untargeted salt”. (*LA* light-acclimated, *SA* shade-acclimated, *0, 50 and 150 mM* NaCl concentration)

Feruloyl putrescines could hardly be detectable in leaves or stems (Fig. 16). In the leaves, the high levels of isoscoparin-O-rhamnosideand 3-O-p-coumaroylquinic acid were still detectable in the LA plants; however, the effects of salt were not obvious. In contrast to the 3-O-p-coumaroylquinic acid, in the case of coumaroylcitric acids, this trend is not visible. Its reason can be that in the case of hydroxycitric acid derivatives, increasing salt concentration led to a decreasing trend, and its level was the lowest in the LA plants. In the stem, many investigated compounds were close to the detection limit, and the other detected compounds did not show remarkable salt or light responses (Fig. 16). In the roots, feruloyl putrescines were in the highest amount in the LA 0 mM plants, but they decreased under saline conditions. In contrast to these, 3-O-p-coumaroylquinic acid shows interesting salt responses in the roots, depending on the light conditions during the cold acclimation period: similarly to the leaves, in LA plants salt induced higher amount than in SA plants. This indicates that the effects of light could be detected again in the roots. Pinellic acid also dominated in the roots; however, its salt response was not obvious (Fig. 16).

Overall, PCA and analysis of individual metabolites show that the response to salt stress is not manifested by isolated compound-level changes, but by organ-specific, yet coordinated metabolic reprogramming that affects central carbon, nitrogen, and redox processes. Cold pre-treatment, especially in light, significantly modified this response pattern, in several cases reducing salt-induced accumulations (e.g., sugars, amino acids, polyamines, jasmonate signalling), while light-dependent activation of certain defence and phenylpropanoid pathways was maintained. All these observations suggest that cold acclimation in light prepares the plant for subsequent stress through integrated, whole-plant metabolic and transcriptional reprogramming.

## Discussion

### Light as a key determinant of effective cold acclimation in maize

In an earlier study, we have shown that cold acclimation can enhance the cold tolerance of maize, and light plays an important role in its development (Szalai et al. 2018). In that work, we have shown that exposure to low temperature in the light may increase lipid peroxidation, reflected by elevated MDA accumulation in leaves, owing to enhanced photooxidative stress. Nevertheless, this oxidative challenge appears to contribute to the establishment of cold acclimation, indicating that a limited degree of photodamage may be the cost of acquiring increased freezing tolerance. We have also characterized the main changes at the gene expression level using a detailed microarray analysis. Carrying out more detailed gene expression and metabolomics analyses, the present work is partly a continuation of that work, and partly a complement to it by answering a new question of how cold acclimation under different light conditions may affect the responses of young maize plants to the presence of another abiotic stressor, elevated salt concentration. It must also be mentioned that although previous stress exposure clearly modified the subsequent response to salinity, the present experimental design does not allow discrimination between a persistent acclimated physiological state and stress memory in the strict mechanistic sense, as salt stress was applied immediately after the cold acclimation period. Therefore, our results are interpreted as evidence that light-dependent cold acclimation establishes organ-specific metabolic and transcriptional reprogramming that shapes subsequent salt stress responses.

The slight decrease after cold or salt treatments in the F_v_/F_m_ parameter indicated that neither cold acclimation nor saline stress caused substantial irreversible damage to the photosynthetic apparatus, similarly as previously reported (Janda et al. 1998; Zahra et al. 2022). However, the pronounced reduction in the light-adapted quantum yield (YII) and the parallel increases in the quenching parameter Y(NPQ) under high (150 mM) salinity, especially in cold-acclimated plants, suggest that cold treatment sensitized the photosynthetic apparatus to subsequent osmotic or ionic stress. This may reflect the activation of downregulation processes, in order to protect the photosynthetic apparatus showing a trade-off between enhanced photoprotective capacity and reduced photosynthetic efficiency, which has been reported in stress-primed plants (Hamow et al. 2024; Chang et al. 2025; Sahoo et al. 2025; Ressaissi et al. 2025).

The PCA analyses focusing on the primary metabolites with or without treatment at relatively high (150 mM) salinity demonstrated consistent treatment-induced changes in metabolic profiles in every organ, suggesting systemic but organ-dependent physiological reprogramming. In our previous work, we have already described the main transcriptional changes during cold acclimation (Szalai et al. 2018). In order to better understand the light-driven cold-related signalling processes in young maize plants, further stress-related genes were also analysed.

The COLD1 gene has already been shown to play a key role in cold tolerance in rice: a single nucleotide mutation in it is responsible for the improved cold tolerance of japonica compared to indica rice (Ma et al. 2015). Under the present experimental conditions, the highest *ZmCOLD1* expression values were obtained in the leaf, which is most exposed to potential oxidative stress. However, the used low-temperature treatments did not increase, but in some cases, decreased the expression level. This does not exclude its role in the cold acclimation processes. Other studies have reported a transient increase, but only during the initial phase of cold treatment (Jin et al. 2021). The response also appears to depend on the temperature applied and the prevailing light conditions. Further targeted and detailed studies are, therefore, needed to better understand the precise role and mode of action of this gene, which appears to play an important role in cold responses.

The *COP1* and *HY5* genes play a role in many plant signalling processes, as it has been shown, for example, in the case of COP1 under endoplasmic reticulum or cold stresses (Kang et al. 2021; Bian et al. 2022), or in the case of HY5, under cold stress (Bian et al. 2022). In our case, both cold acclimation and severe cold stress led to a drastic decrease in their expression levels, but the effect of light was also detectable, supporting the idea that they also play a role in light regulation (Nawkar et al. 2017; Jiang et al. 2020). The similar behaviour of COP1 and HY5 is not surprising, because they are in a regulatory hub. Although COP1 can inhibit HY5, it also inhibits genes that inhibit HY5 (Bian et al. 2022). Besides stress responses, COP1 and HY5 may also regulate photomorphogenesis; therefore, their downregulation under stress conditions can also partly be linked to growth inhibition and reallocation of resources toward the defence processes (Gangappa and Botto 2016; Huai et al. 2020). Similar to these genes, low temperatures led to a decrease in the *PAO1* gene, playing role in the catabolism of polyamines. Besides cold, increasing salinity also downregulated the *PAO1* expression level. This supports the view that polyamine metabolisms may play a key role in the light-regulated stress responses (Pál et al. 2021), and are in agreement with the finding that silencing cytoplasmic PAOs may increase salinity tolerance in plants (Sagor et al. 2016).

An interesting observation was that *ZmPAO1* expression remained comparatively high in stems under several cold acclimation treatments, despite the general reduction observed in leaves and roots. Polyamine oxidases contribute to polyamine turnover while generating H₂O₂, thereby linking polyamine metabolism with redox signalling. Although our data do not allow conclusions regarding the functional role of stem tissues, this expression pattern may indicate that the stem contributes to the coordination of systemic stress responses by maintaining redox-related signalling during cold acclimation and subsequent stress exposure.

The role of polyamines in the light-regulated cold acclimation processes can also be detected in the putrescine levels in the leaves or stem, where substantial increase was only observed in the LA and LA-Cold plants. Interestingly, the terminal catabolite product of higher polyamines, DAP followed the pattern of PUT. Furthermore, in the root, light-dependent increase was detected not in the PUT, but in the other polyamine levels. In the case of salt stress, the picture was different. Here, remarkable concentration-dependent increases in the level of higher polyamines were observed. However, the lack of increase in spermidine and the reduced DAP accumulation in LA plants indicate lower activation of polyamine turnover related to the polyamine cycle (Pál et al. 2015). This cycle generates H₂O₂, which may contribute to stress signalling (Moschou et al. 2008); thus, reduced catabolic activity in LA plants may suggest reduced stress perception. The different responses of the polyamine levels to the cold and saline conditions in the different organs are not surprising, because cold “attacks” every organ with a light-driven oxidative signal from the leaves, and salt mainly “attacks” via the roots. Nevertheless, our results are consistent with the generally accepted roles of polyamines in membrane stabilization, ion channel regulation and scavenging of reactive oxygen species (Pál et al. 2015). Although these physiological functions were not measured directly in the present study, the observed metabolite profiles are consistent with such protective roles reported previously. Furthermore, the induction of feruloyl putrescines exclusively in roots further suggests a specialized strategy combining polyamine and phenylpropanoid metabolism to reinforce cell walls and regulate ion fluxes under stress.

In contrast to these, cold treatments could lead to an increase in the expression levels of the *PMP3-1* and *ZmSWEET1a* genes. Similarly, *PMP3-5* also generally decreased under saline conditions, especially at higher concentrations. PMPs may play a role in responses to osmotic stresses induced by drought, cold, or high salinity (Fu et al. 2012). In an earlier study, the different *PMP* genes were differentially responsive to abiotic stresses. Investigations on the molecular mechanism in *ZmPMP3-1* over-expressing transgenic plants could also aid in the elucidation of the regulatory mechanism of PMP3 in response to abiotic stresses and maintenance of ion homeostasis in plants (Fu et al. 2012). Accordingly, the observed regulation of PMP genes may reflect adjustments associated with maintaining membrane potential and cellular ion balance during combined cold and salt stress, although ion fluxes were not determined in the present work. *ERF* genes also play a multiple role in plant development and stress acclimation, including regulation of cold and salt stress responses (Zhang and Huang 2010; Yang et al. 2014). Certain key phytohormones, such as ABA and MeJA may also regulate their expression levels to mediate stress adaptation processes (Rubio et al. 2019; Niu et al. 2025). Under the present conditions, cold-induced upregulation of the *ERF33* gene only occurred in the leaves, suggesting its specific role in this organ, and this induction was also light-dependent, which may also contribute to the light-induced cold tolerance. All in all, the present results also support our earlier conclusion (Szalai et al. 2018) that photoinhibition-associated excitation pressure in the photosynthetic electron transport rate, rather than being merely damaging, serves as an essential signal for the induction of cold tolerance mechanisms. Besides the above-mentioned ones, *ZIP1* gene also generally decreased under saline conditions, except in the leaf of previous cold-acclimated plants, where an increasing trend was observed with the salt concentration. In contrast to this, *DREB80* generally increased at low concentration of salt in the SA-50 plants. DREB family is among most important transcriptional factors, being also involved in salt stress responses (Hou et al. 2022), and it seems that its regulation is also indirectly affected by the combination of temperature, light and salt.

### Metabolomic changes under cold and/or saline conditions

In agreement with earlier findings (Thomashow 1999; Szalai et al. 2018), in the case of sugar or sugar alcohol compounds, a pronounced increase occurred mainly in the leaves after exposure to acclimating temperatures in the light. Interestingly, the extreme low temperatures rather decreased the sugar contents in the leaves but increased them in the stem and root. The different accumulations between the organs can most probably be explained by the reduced synthesis in the leaves and the active transport towards the other organs (Sowiński et al. 1999; Burnett and Kromdijk 2022). In the shade cold-acclimated plants, these changes were much less pronounced. As an osmotic stress, it is not surprising that high salinity also causes an increase in the sugar levels, as it was observed in the control plants. Interestingly, this increase could not be generally observed in the cold-acclimated plants. Since soluble sugars contribute not only to osmotic adjustment but also to membrane stabilization and protection of cellular structures under saline conditions, the reduced accumulation may have limited the capacity for osmotic compensation in these plants. This interpretation is consistent with the fluorescence measurements indicating a stronger decline in photosynthetic performance, although direct measurements of osmotic potential or ion balance would be required to confirm this mechanism.

In general, at low temperatures, amino acids behaved similarly to the sugars, and here the light-induced changes were even more pronounced. Besides serving as compatible solutes, increased amino acid pools may also provide metabolic flexibility by supporting nitrogen remobilization and the synthesis of protective metabolites during stress recovery. The roles of different amino acids under stress conditions have been widely studied. Proline is one of the best characterised ones, playing both osmoprotectant and signalling roles (Szabados and Savouré 2010). Besides these, they may also scavenge reactive oxygen species, or may act as precursors for other protective metabolites, such as, among others, glycine betaine (Khalil et al. 2021). Interestingly, in most cases, oxoproline was the most abundant amino acid-related compound (Supplementary Table S4). As part of the glutathione cycle, oxoproline is formed during the regeneration of glutathione. Then oxoproline is converted back to glutamate, from which new glutathione can be built, in order to maintain the redox balance of the cell. Besides this role, oxoproline also appears as a by-product of proline synthesis or degradation under certain conditions, so indirectly, it may also contribute to the fine-tuning of the stress response, and it may also serve as a signal under stress conditions (Ito and Ohkama-Ohtsu 2023). Not only low temperatures, but high salinity also increased the amino acid levels. However, in contrast to cold, this increase was less pronounced in the light-acclimated than in either the control or in the shade-acclimated ones.

Similarly to the present results, light-dependent root responses have recently been shown in young rice plants, demonstrating the presence of shoot–root signalling pathways involving not only sugars but also hormones and redox signals (Gholizadeh et al. 2024). In the present work, among the plant hormones, the salicylic acid, the JA with its conjugates and ABA were in focus. Salicylic acid is an important plant hormone playing role in both plant stress responses and development (Peng et al. 2021; Li et al. 2025). Although it is known to play a role in several types of stress processes, it responded differently to cold and salinity conditions under the current circumstances. While at low temperatures, a decrease was observed both in the leaves and roots, it slightly increased at severe cold in the stem. In contrast to this, salt caused an increase mainly in the root, but changes also occurred in the leaves and stem. Since previous literature data give a very varied picture of the changes in salicylic acid under stress (Pál et al. 2020; Wang et al. 2013), we can assume that the amount of salicylates is regulated in a very complex manner, including different synthesis pathways and compartmentalization processes. It most probably plays a regulatory, and not a central role in the acclimation to these stressors. Thus, different conditions, genotype, and external factors play a major role in the development of the amount. In contrast to this, another plant hormone jasmonic acid/Ile conjugate, together with the free form, jasmonic acid showed a substantial decrease under cold conditions, and this was also visible after several days, during the salt stress period. Its reason is unknown, because other studies usually report an increase in plants during the cold period (Ding et al. 2022; Kim et al. 2021). In contrast to this, in accordance with earlier findings (Song et al. 2021), an increase was shown under saline conditions in the stem. Furthermore, the fact that cold and high salinity may share certain signalling components are clearly demonstrated by the changes in the abscisic acid levels: it showed a stress level-dependent increase under both cold and saline conditions. The parallel changes in the abscisic acid level with its catabolites indicate enhanced biosynthesis rather than reduced metabolism.

The light-dependent accumulation of ferulic acids, flavonoids and coumaroyl derivatives under cold acclimation supports the hypothesis that structural reinforcement and antioxidant protection are integral components of maize cold responses. Feruloylation of cell wall polymers can enhance mechanical strength and reduce membrane leakage, while flavonoids contribute to ROS scavenging and photoprotection. Although the quantitative changes in hydroxycinnamic acids and hydroxybenzoic acids were not uniform, several key phenylpropanoid derivatives showed treatment-specific changes, suggesting that the effect of pre-cold treatment can also be detected in secondary metabolic changes during salt stress. Chlorogenic acids may contribute to membrane protection and ROS scavenging, suggesting that LA plants prefer to enhance redox-buffering phenolics. The salt-induced, concentration-dependent increase in chlorogenic acids in leaves, especially in plants pre-cold-acclimated to light, is consistent with the role of these compounds as antioxidants and modulators of cellular redox balance during abiotic stress (Sharma et al. 2019). Similarly, the salt-induced increase in ferulic acid levels was more pronounced in the roots of plants previously cold-acclimated in the light, suggesting an enhancement of cell wall-related defence mechanisms. Ferulic acid derivatives are involved in the cross-linking and lignification of cell wall polysaccharides, which are known to limit ion leakage and mechanical damage under saline conditions (Burr and Fry 2009). These changes are consistent with enhanced structural adaptation at the soil–plant interface, where ionic stress is first perceived. However, since cell wall composition and ion leakage were not measured directly, this interpretation should be regarded as a physiological hypothesis supported by the metabolomic data.

## Conclusions and perspectives

Overall, our results indicate that low-temperature acclimation alters transcriptional and metabolic pathways in maize in a way that has consequences for subsequent salt stress responses. Furthermore, light during the cold-acclimation period may also fundamentally modify the acclimation processes. Some differences may persist for several days after cold acclimation, while others were reduced. The observed metabolic changes should, therefore, be regarded as indicators of altered physiological states rather than direct evidence of specific mechanisms affecting membrane stability, ion homeostasis or osmotic adjustment, which will require targeted physiological analyses in future studies. Light-induced metabolic changes occur not only in leaves but also in stem and roots indicating a systemic acclimation mechanism, not a purely photosynthetic tissue phenomenon. It must also be mentioned that stress sensitivity measured in individual organs does not necessarily reflect the sensitivity of the whole plant.

In general, the diverse metabolic changes occurring during cold acclimation, especially under light, suggest that some acclimation-induced changes remained detectable during the subsequent salt treatment and were associated with improved metabolic stability. Hormonal responses also support this view, with strong ABA-driven salt responses maintained, while jasmonic acid- and salicylic acid-related changes were moderated in pre-acclimated plants, suggesting a shift towards energy-efficient osmotic regulation. However, in light-acclimated plants, phenylpropanoid compounds showed a greater induction, consistent with enhanced antioxidant and structural defences. The responses were organ-specific, with roots showing a clear adaptation of polyamines and phenolic conjugates, highlighting coordinated but differentiated systemic regulation. Our results indicate that metabolic responses to salt stress are highly organ-specific, yet systemically coordinated. In leaves, pathways related to photosynthesis, redox homeostasis, and osmoprotection were dominant, while active metabolic reprogramming in the stem indicated a central role for carbon–nitrogen rearrangement and signalling processes, while in roots, changes in respiration, nitrogen storage, and polyamine metabolism were more dominant. This organ specificity underscores the importance of integrated, whole-plant approaches.

The results show that although the applied physiological parameters (MDA in root, fluorescence induction in the leaves) could only demonstrate differences between the acclimated and non-acclimated plants, many metabolomic parameters could clearly differentiate between light- and shade-acclimated plants as well. Therefore, as a continuation of the experiments, it would be worthwhile to perform more detailed stress physiological studies in the future, on one hand, with multiple salt concentrations, sampling at multiple times, and on the other hand, using other stressors. Future work combining functional genetics and flux-based metabolomics will be essential to disentangle causal regulatory mechanisms and exploit acclimation processes in maize breeding programs.

## Supplementary Information

Below is the link to the electronic supplementary material.

Supplementary file1 (PDF 247 KB)

Supplementary file2 (XLSX 19 KB)

Supplementary file3 (DOCX 19 KB)

Supplementary file4 (XLSX 21 KB)

Supplementary file5 (XLSX 194 KB)

## Data Availability

All data generated or analysed during this study are included in this published article and/or its supplementary information files.
