# Supplementary material for: Light-defined cold acclimation reprograms organ-specific metabolic and transcriptional landscapes for salt stress responses in maize
Source: Plant Cell Rep. 2026 Oct 5;45(10):320. doi: 10.1007/s00299-026-04008-z (PMC13638696; doi:10.1007/s00299-026-04008-z)
Supplement: Supplementary file 1 [file 299_2026_4008_MOESM1_ESM.pdf]

Supplementary Figure

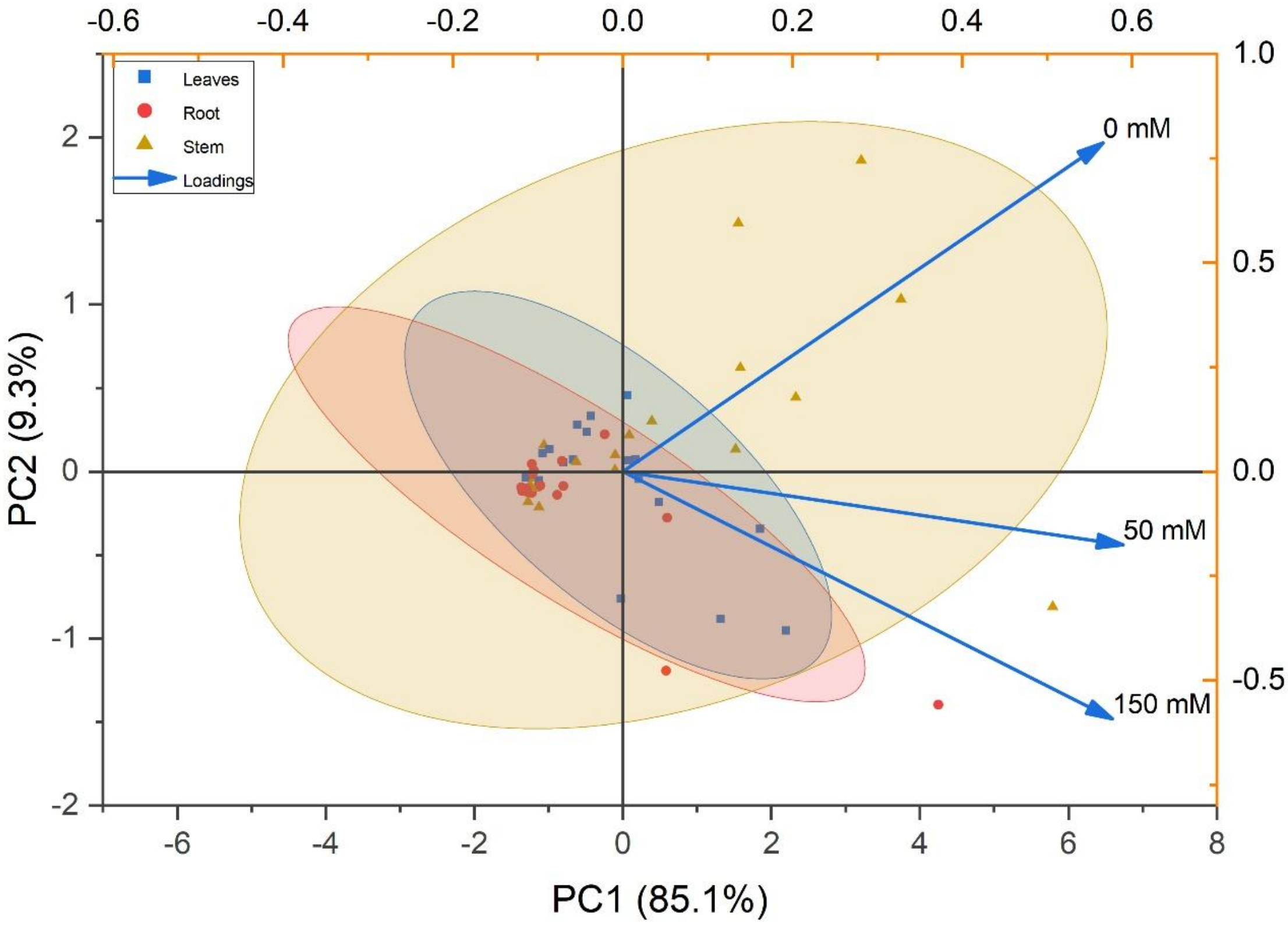

Supplementary Figure_S1. Principal component analysis (PCA) of organ-specific changes in gene expression in maize plants exposed to different salt concentrations. PCA was performed using the changes in the expression of the selected genes in leaves, stems, and roots. Individual samples are shown by symbols according to the plant organ: leaves (blue squares), roots (red circles), and stems (yellow triangles). The arrows indicate the loadings associated with the different salt treatments (0, 50, and 150 mM NaCl). The percentages in parentheses indicate the proportion of total variance explained by each principal component. Ellipses represent the distribution of samples within each organ.
