# Supplementary material for: Light-defined cold acclimation reprograms organ-specific metabolic and transcriptional landscapes for salt stress responses in maize
Source: Plant Cell Rep. 2026 Oct 5;45(10):320. doi: 10.1007/s00299-026-04008-z (PMC13638696; doi:10.1007/s00299-026-04008-z)
Supplement: Supplementary file 2 [file 299_2026_4008_MOESM3_ESM.docx]

**Supplementary Table S2** List of the 31 assigned known and tentative secondary metabolites detected in the maize samples. Identification levels are indicated according to Schymanski et al. (2014). ‘RT’, retention time; ‘CCS’, collisional cross section; ’*’, sodiated adduct.

| **Component name** | **Formula (neutral composition)** | **Observed RT (min)** | **Theoretical *m/z* [M+H]^+^** | **Experimental *m/z* [M+H]^+^** | **Difference, ppm** | **CCS in ESI ‘+’ mode, (Å)^2^** | **Theoretical *m/z* [M-H]^-^** | **Experimental *m/z* [M-H]^-^** | **Difference, ppm** | **CCS in ESI ‘-’ mode, (Å)^2^** | **Identification level** |
| --- | --- | --- | --- | --- | --- | --- | --- | --- | --- | --- | --- |
| Aminoacid derivative_1 | C15H22N2O4 | 2.34 | 295.1647 | 295.1648 | 0.34 | 168.83 | - | - | - | - | 3 |
| Aminoacid derivative_2 | C14H20N2O4 | 2.47 | 281.1496 | 281.1495 | -0.28 | 167.04 | - | - | - | - | 3 |
| Neochlorogenic (3-caffeoylquinic) acid | C16H18O9 | 2.81 | - | - | - | - | 353.0878 | 353.0872 | -1.73 | 171.73 | 1 |
| Isoferuloyl putrescine | C14H20N2O3 | 3.26 | 265.1547 | 265.1550 | 1.24 | 161.76 | - | - | - | - | 2a |
| 2-O-Caffeoylhydroxycitric acid | C15H14O11 | 3.74 | - | - | - | - | 369.0463 | 369.0461 | -0.62 | 183.46 | 2a |
| 3-O-p-coumaroylquinic acid | C16H18O8 | 3.76 | - | - | - | - | 337.0929 | 337.0930 | 0.33 | 181.66 | 2a |
| N-feruloyl putrescine | C14H20N2O3 | 4.09 | 265.1547 | 265.1547 | 0.11 | 172.13 | 263.1401 | 263.1398 | -1.22 | 172.51 | 1 |
| Coumaroylhydroxycitric acid | C15H14O10 | 4.22 | - | - | - | - | 353.0514 | 353.0515 | 0.23 | 165.52 | 2b |
| Coumaroylhydroxycitric acid | C15H14O10 | 4.65 | - | - | - | - | 353.0514 | 353.0512 | -0.62 | 178.98 | 2b |
| Feruloylhydroxycitric acid | C16H16O11 | 5.38 | - | - | - | - | 383.0620 | 383.0616 | -0.99 | 187.74 | 2a |
| Vicenin-2 | C27H30O15 | 6.02 | 595.1658 | 595.1661 | 0.59 | 240.76 | 593.1512 | 593.1513 | 0.19 | 239.85 | 2a |
| 13:4+4O fatty acyl hexoside | C19H28O10 | 6.41 | - | - | - | - | 415.1610 | 415.1619 | 2.24 | 194.57 | 2a |
| Isoschaftoside | C26H28O14 | 7.30 | 565.1552 | 565.1547 | -0.85 | 232.80 | 563.1406 | 563.1410 | 0.66 | 231.82 | 2a |
| Chrysoeriol 7-O-glucuronopyranosyl-(1-2)-O-glucuronopyranoside | C28H28O18 | 8.24 | 653.1348 | 653.1343 | -0.83 | 248.81 | 651.1203 | 651.1209 | 0.94 | 243.58 | 2b |
| Rutin | C27H30O16 | 8.36 | 611.1607 | 611.1613 | 1.05 | 236.18 | 609.1461 | 609.1475 | 2.28 | 234.64 | 1 |
| Isovitexin | C21H20O10 | 8.43 | 433.1129 | 433.1130 | 0.18 | 199.52 | 431.0984 | 431.0988 | 1.00 | 202.42 | 2a |
| Isoscoparin-O-rhamnoside | C28H32O15 | 8.80 | 609.1814 | 609.1820 | 0.98 | 240.64 | 607.1668 | 607.1665 | -0.56 | 248.75 | 2a |
| Hydroxycitric acid derivative 1 | C21H22O12 | 13.47 | - | - | - | - | 465.1039 | 465.1041 | 0.54 | 220.51 | 3 |
| Hydroxycitric acid derivative 2 | C21H22O12 | 13.61 | - | - | - | - | 465.1039 | 465.1039 | 0.11 | 222.69 | 3 |
| 9S,10S,11R-trihydroxy-12Z,15Z-octadecadienoic acid_1 | C18H32O5 | 14.25 | - | - | - | - | 327.2177 | 327.2178 | 0.31 | 184.46 | 2b |
| 9S,10S,11R-trihydroxy-12Z,15Z-octadecadienoic acid_2 | C18H32O5 | 14.43 | - | - | - | - | 327.2177 | 327.2178 | 0.31 | 185.37 | 2b |
| Pinellic acid | C18H34O5 | 14.97 | - | - | - | - | 329.2334 | 329.2331 | -0.76 | 187.42 | 2a |
| O-acyl-sugar | C27H32O14 (Na) | 15.04 | 603.1684* | 603.1685* | 0.12 | 235.87 | 579.1719 | 579.1721 | 0.29 | 227.08 | 3 |
| 1-mono-O-acyl-3-O-(alpha-D-sulfoquinovosyl)-glyceride (SQMG) C18:4 | C27H46O12S | 16.07 | - | - | - | - | 593.2637 | 593.2635 | -0.37 | 238.59 | 2a |
